# Coupling of CuO@NiBiO_x_ Catalyzed Glycerol Oxidation to Carbon Dioxide Reduction Reaction for Enhanced Energy Efficiency

**DOI:** 10.1002/anie.202502617

**Published:** 2025-04-17

**Authors:** Thi‐Hong‐Hanh Le, Yong Zuo, Manjunath Chatti, Martina Rizzo, Andrea Griesi, Abinaya Annamalai, Simone Lauciello, Luca Leoncino, Mirko Prato, Silvia Dante, Ilka Kriegel, Giorgio Divitini, Michele Ferri, Liberato Manna

**Affiliations:** ^1^ Istituto Italiano di Tecnologia (IIT) Via Morego 30 Genova Italy; ^2^ Università degli studi di Genova (UniGe) Via Dodecaneso 31 Genova Italy; ^3^ School of Chemistry and Chemical Engineering Chongqing University Chongqing 400044 China; ^4^ Dipartimento di Scienze Applicate e tecnologia (DISAT) Politecnico di Torino Corso Duca degli Abruzzi 34 Torino Italy

**Keywords:** CO_2_ reduction, Electrochemistry, Energy saving, Glycerol electrooxidation, Paired electrosynthesis

## Abstract

Glycerol electrooxidation reaction (GEOR) is a promising alternative to the oxygen evolution reaction (OER) in electrolyzers, overcoming the inherent challenges of high energy demand and low‐value output of water oxidation. Here, we designed a non‐noble metal‐based electrocatalyst (CuO@NiBiO_x_, CNBO) for selective and efficient GEOR. The CNBO catalyst demonstrated high selectivity and achieved nearly 100% GEOR Faradaic efficiency (FE), 80%–90% of which is conveyed into formic acid (FA). Bismuth incorporation modified the structure of the mixed oxide, increasing the surface concentration of Ni(III) species and enhancing the GEOR activity. In situ studies confirmed the formation of NiOOH, which is identified as the active site for GEOR and suggests an indirect GEOR mechanism. This study demonstrates the potential of GEOR to replace OER in Carbon dioxide reduction reaction (CO_2_RR) electrolyzers. Depending on the selected CO_2_RR catalyst (Ag or Sn), we could obtain either an easy‐to‐separate mixture of high‐added value products (CO and FA) or a single product (FA) with FE_FA_ > 85% at both electrodes. Moreover, we demonstrate that replacing OER with GEOR in a CO_2_RR‐electrolyzer can save up to 25% of the electrolysis energy input, while the co‐production of FA at both electrodes halves the energy per mole required for its electrosynthesis.

## Graphical abstract

A CuO_x_@NiBiO_x_ core‐shell‐like catalyst is synthesized. Thanks to the Bi presence, the electronic structure of Ni is tuned, leading to better catalytic performance for glycerol oxidation compared to both CuO_x_ and CuO_x_@NiO_x_ catalysts. Additionally, coupling glycerol oxidation with CO_2_ reduction significantly reduces the operational voltage and the specific energy required for formate production.

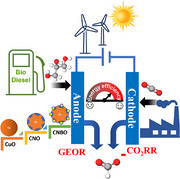

## Introduction

With the growing awareness of the severity of the global energy crisis and the environmental damage caused by the extensive use of fossil fuels, together with the high carbon intensity of manufacturing, there is a strong push toward sustainable development.^[^ ^1^ ^]^ This shift envisions the adoption of cleaner, renewable energy sources. Among the promising solutions, biofuels derived from animal fats and vegetable oils have emerged as a viable alternative.^[^ ^2^ ^]^ These biofuels offer a way to mitigate the reliance on fossil fuels, lower carbon emissions, and promote environmental sustainability, making them an increasingly important component of the global transition to cleaner energy.^[^ ^3^ ^]^ Along with the increase in biofuel production, glycerol is generated as a byproduct of biodiesel production, constituting ∼10 wt%.^[^ ^4^ ^]^ Global biodiesel production in 2022 was 52 million tons, meaning that more than 5.0 million tons of glycerol was produced as a by‐product.^[^ ^5^, ^6^ ^]^ The generation of the unprecedented surplus in glycerol has become an issue in recent years, increasing the price of biofuel.^[^ ^7^ ^]^ Therefore, converting glycerol into a high‐value product would generate value, remove an undesired component, and reduce the total cost of biofuel.

The glycerol electrooxidation reaction (GEOR) has thus been investigated as a green route toward sought‐after carbon‐based products.^[^ ^8^ ^]^ Indeed, glycerol can be oxidized to produce high added‐value compounds, such as dihydroxyacetone, tartronic acid, glycolic acid, and formic acid (FA), which all have vital applications in the cosmetic and pharmaceutical industry.^[^ ^9^, ^10^ ^]^ Thanks to its favorable thermodynamics (e.g., the standard oxidation potential for glycerol oxidation to FA is 0.69 V vs. RHE, much lower than the 1.23 V vs. RHE of OER^[^ ^11^ ^]^) and the possible generation of high‐added value products, GEOR is a promising anodic alternative to the typical OER, and drive up energy efficiency and profitability of an electrolytic process.^[^ ^10^ ^]^ However, GEOR faces several criticalities, with selectivity being one of the most pressing issues. Indeed, the glycerol electrooxidation mechanism is complex, with multiple intermediates and branched reaction pathways that can lead to a scattered product distribution.^[^ ^12^ ^]^ Also, deep GEOR can even result in complete oxidation and thus CO_2_ emission.^[^ ^13^ ^]^ Therefore, developing a catalyst that preferentially promotes GEOR over OER while also achieving selectivity toward a specific added‐value product represents an open challenge.

Noble metal‐based catalysts have been extensively investigated for GEOR and were found to exhibit good catalytic activities.^[^ ^14^ ^]^ However, their use presents several drawbacks. First, noble metals are scarce and expensive, thus raising concerns about their cost‐effectiveness and sustainability in large‐scale applications (as for other electrocatalytic research fields, e.g., HER). Additionally, noble metals often lead to a complex mixture of products, requiring extensive downstream purification that adds to operational costs. For example, Pt‐based catalysts usually produce a mixture of glyceraldehyde, glycerate, lactate, tartronate, glycolate, oxalate, formate, and dihydroxyacetone.^[^ ^15^, ^16^ ^]^ Another significant issue is that the surfaces of noble metal catalysts are prone to poisoning by glycerol and its intermediate products, thus reducing their effectiveness over time.^[^ ^17^, ^18^ ^]^ Moreover, under specific applied potentials (i.e., 0.95 V vs. RHE for Pt), an inert oxide layer can be formed on the catalyst, leading to deactivation.^[^ ^19^ ^]^ Overall, while noble metals demonstrate promising catalytic abilities, these limitations pose substantial barriers to practical applications.

Against this backdrop, developing a non‐noble metal‐based catalyst with high GEOR selectivity is a fundamental stepping‐stone. Transition metal‐based catalysts have drawn researchers’ attention thanks to their activity in several electrochemical reactions.^[^ ^20^, ^21^, ^22^ ^]^ Among them, catalysts based on nickel, a cost‐effective, and abundant element, have been widely and successfully investigated in oxidation reactions.^[^ ^23^, ^24^, ^25^ ^]^ Generally, Ni‐based catalysts undergo surface reconstruction leading to the generation of Ni(III) species (in the form of nickel oxyhydroxide, NiOOH), the main active site for oxidations.^[^ ^26^, ^27^ ^]^ However, the use of Ni‐based catalysts in organic molecule electrooxidation has been limited by several factors: OER competition, poor electrical conductivity, and instability under high current conditions.^[^ ^4^, ^28^, ^29^ ^]^ To overcome these challenges and enhance performance, the modification of Ni‐based catalysts has been pursued, for example, by incorporating co‐catalysts. The combination of Ni‐based materials with Cu was reported to promote robust catalytic performance by enhancing the electrical conductivity and facilitating the generation of Ni(III) active sites.^[^ ^30^, ^31^, ^32^ ^]^ Besides, the incorporation of OER‐inert copper species is demonstrated to help suppress the competition of OER, widening the working potential range.^[^ ^33^ ^]^ Another challenge usually encountered by Ni‐based catalysts in GEOR is deep oxidation to CO_2_.^[^ ^34^, ^35^ ^]^ Interestingly, bismuth addition to noble metal‐based catalysts has been demonstrated to prevent C─C bond cleavage, thus minimizing the generation of carbon monoxide and dioxide in GEOR.^[^ ^36^, ^37^ ^]^ The effect of Bi on Ni‐based catalysts for GEOR was investigated by Houache and his group.^[^ ^4^ ^]^ Despite the unclear effect exerted by Bi itself on the Ni‐based active sites, the addition of Bi_2_O_3_ to Ni(OH)_2_ not only improved catalytic performance compared to monometallic catalyst by accelerating the reaction kinetics and lowering the GEOR onset potential but, consistently with what was observed on noble metals, also inhibited carbon chain cleavage, thereby minimizing CO_2_ production.

Inspired by these studies, we rationally designed and synthesized a mixed oxide CuO@NiBiO_x_ (CNBO) core‐shell catalyst for highly efficient GEOR, able to suppress parasitic water oxidation. CNBO exhibited good catalytic activity and stability over a practical 20‐h electrolysis period at the current density of 50 mA cm^−2^, achieving near‐unit GEOR Faradaic efficiency (FE) and exhibiting high selectivity for formic acid production. A combination of photoemission spectroscopy and electrochemistry was employed to investigate the electronic structure underlying the superior performance of CNBO compared to the control samples (CuO@NiO_x_ and CuO). The addition of bismuth to CNBO enhanced the catalyst performance while also suppressing the competing water oxidation. Thanks to in situ Raman experiments, we could probe the dynamic reconstruction of CNBO catalyst during the reaction and prove the indirect oxidation mechanism of GEOR. Finally, we extended the scope of GEOR by integrating it with the carbon dioxide reduction reaction (CO_2_RR) in a flow electrolyzer cell, aiming at producing easy‐to‐separate mixtures or cogenerating the same product from both the anodic and cathodic sides.^[^ ^10^ ^]^ This new coupling setup allowed us to reduce the operational energy up to 25% compared to the traditional OER‐CO_2_RR electrolyzers. Furthermore, coproducing formic acid at two electrodes significantly reduces the specific energy required for formic acid production by 52.5%.

## Results and Discussion

### Catalyst Synthesis and Characterization

**Figure 1:**
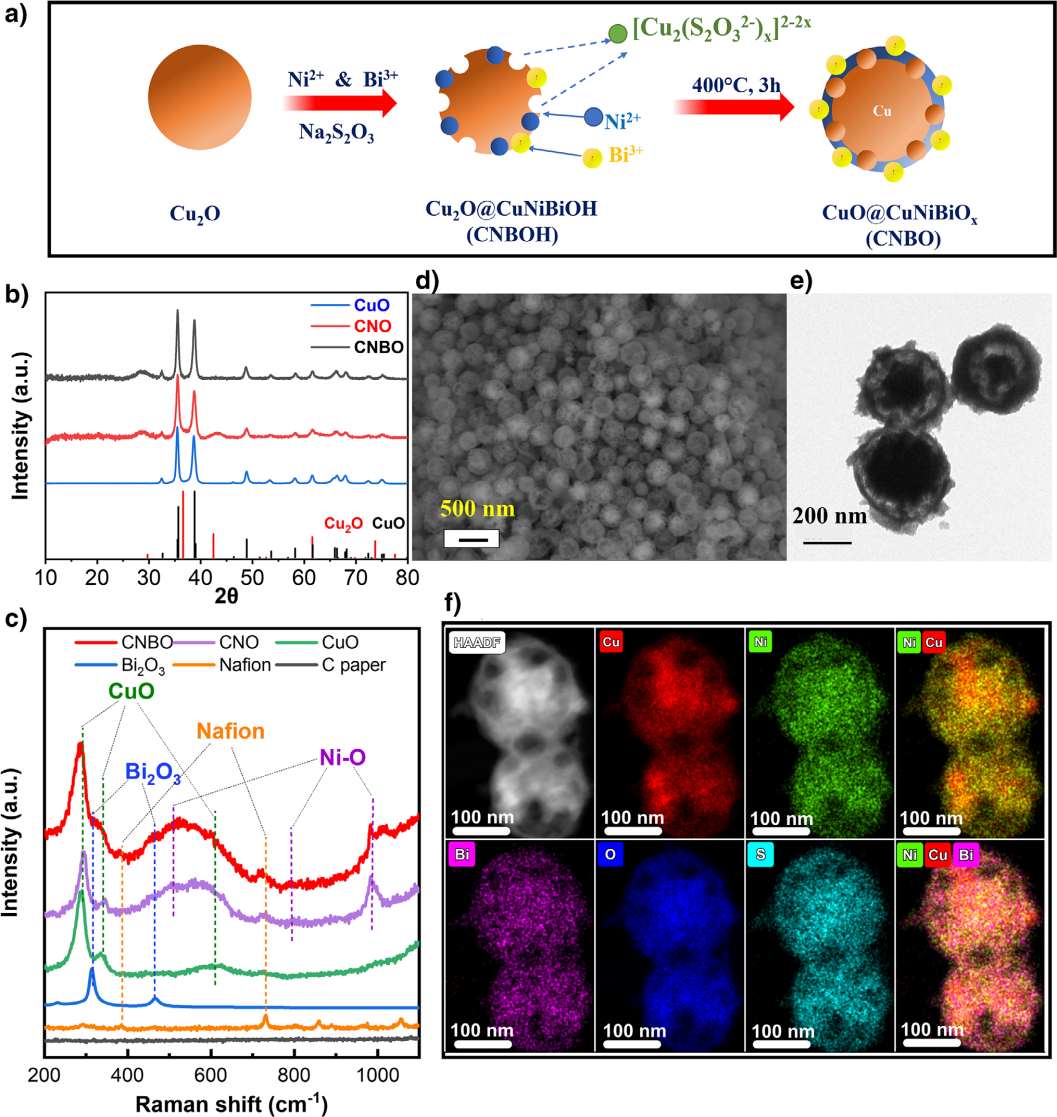
Synthesis and characterization of GEOR electrocatalysts. a) Schematic depiction of CNBO synthetic route; b) XRD pattern of CuO (Cu_2_O spheres after annealing), CNO, and CNBO; c) Raman spectra collected on the actual pristine electrodes (CNBO, CNO, and CuO) and related substrates (here reported as “blanks”); CNBO imaging: d) SEM, e) Bright field TEM, and f) HAADF and EDS elemental maps.

At first, copper (I) oxide (Cu_2_O) spheres with a size distribution between 150 and 400 nm were synthesized according to a simple procedure involving the chemical reduction of a Cu precursor with ascorbic acid (Figure S1 and related details in Supporting Information). A thorough characterization confirmed the spherical morphology and chemical composition of Cu_2_O (Figure S2). CuO@NiBiO_x_ (CNBO), a mixed Cu–Ni–Bi oxide with a core‐shell morphology was then synthesized by a coordinated etching and precipitation method (CEP) using the Cu_2_O spheres as hard templates (Figure 1a). Briefly, Ni^2+^ and Bi^3+^ ions were first adsorbed onto the surface of Cu_2_O spheres; then, the CEP was performed by adding a mild reducing agent (i.e., S_2_O_3_^2−^). The partial surface dissolution of the Cu_2_O spheres, operated by the reducing agent (S_2_O_3_^2−^ ions), yields a soluble copper complex while releasing OH^−^ ions. This local alkalinization at the surface of Cu_2_O spheres resulted in the formation of a core‐shell structure by promoting the precipitation of Ni and Bi hydroxides. The sample was finally annealed at 400 °C in the air for 3 h, by which native hydroxides were converted to oxides. Full details on the syntheses and optimization of CNBO are provided in the Supporting Information (Figures S3 and S4 and related discussion). We also synthesized CuO@NiO_x_ (CNO), following the same procedure described above for CNBO but without adding Bi^3+^ to the reaction (Figure S5). Additionally, we annealed Cu_2_O spheres to obtain the bare CuO. These two samples were used as controls.

The synthesized materials underwent an in‐depth physical–chemical characterization. At first, the chemical composition of CNBO was assessed by inductively coupled plasma optical emission spectroscopy (ICP‐OES) (Table S1). All the expected elements (Cu, Ni, and Bi) were detected, confirming the successful formation of Ni and Bi hydroxides on the Cu_2_O template and the efficiency of the CEP synthesis. X‐ray diffraction (XRD) patterns (Figure 1b) of CuO formed upon Cu_2_O annealing, CNO, and CNBO all exhibit only CuO and Cu_2_O phases. This might be caused by the predominant Cu content in CNO and CNBO and/or the amorphous nature of the Ni–Bi shell. Raman spectroscopy measurements (Figure 1c), carried out on pristine electrodes, further supported the ICP data. All samples exhibit peaks at 290, 333, and 621 cm^−1^, mainly corresponding to the A_g_, B_g_, and B_g_ vibrations of CuO.^[^ ^38^, ^39^ ^]^ Most importantly, the presence of broad peaks around 500, 780, and 1000 cm^−1^ clearly indicates the presence of nickel oxide (in the form of NiO).^[^ ^40^, ^41^ ^]^ Similarly, the presence of peaks at 318 and 460 cm^−1^ indicates the presence of Bi in the form of Bi_2_O_3_ in the CNBO composite. A preliminary investigation of the morphology (Figure 1d,e) indicated a core‐shell structure for CNBO, with a mixed Ni‐Bi oxide forming the outer shell (Figure S7 and related discussion). However, an in‐depth investigation by STEM‐HAADF and EDS mapping (Figure 1f) demonstrated that CNBO has indeed a core‐shell‐like morphology but with the shell being more accurately described as a nanocage. High‐resolution STEM‐EDS analysis evidenced a more homogeneous distribution of the constitutive elements than what was expected for a proper core‐shell structure, seemingly indicating the presence of copper in the outer nanocage. Consistently, X‐ray photoelectron spectroscopy (XPS, Figure S7) detected Cu in both CNO and CNBO samples, corroborating the STEM‐EDS mapping and confirming the presence of Cu in the outer region of the particles. The high‐resolution XPS spectra in the Ni 2p region evidenced interesting differences between samples in the oxidation state of the constitutive elements. As detailed in the following section, in CNO, Ni is present as Ni^2+^ and Ni^3+^, whereas only Ni^3+^ is found in CNBO. Such peculiarity suggests that bismuth, detected in CNBO as pure Bi^3+^, modulates the electronic state of nickel, stabilizing its oxidized state.

### Three‐Electrode Cell Electrochemical Testing

To evaluate the GEOR electrocatalytic performance of the synthesized materials, a basic electrochemical study under a three‐electrode configuration was carried out in an H‐cell. An aqueous 1 M KOH solution, with or without glycerol (0.1 M) was employed as an electrolyte. Additional details, comprising a depiction of this setup, can be found in the Supporting Information (Figure S8 and related paragraph).

**Figure 2:**
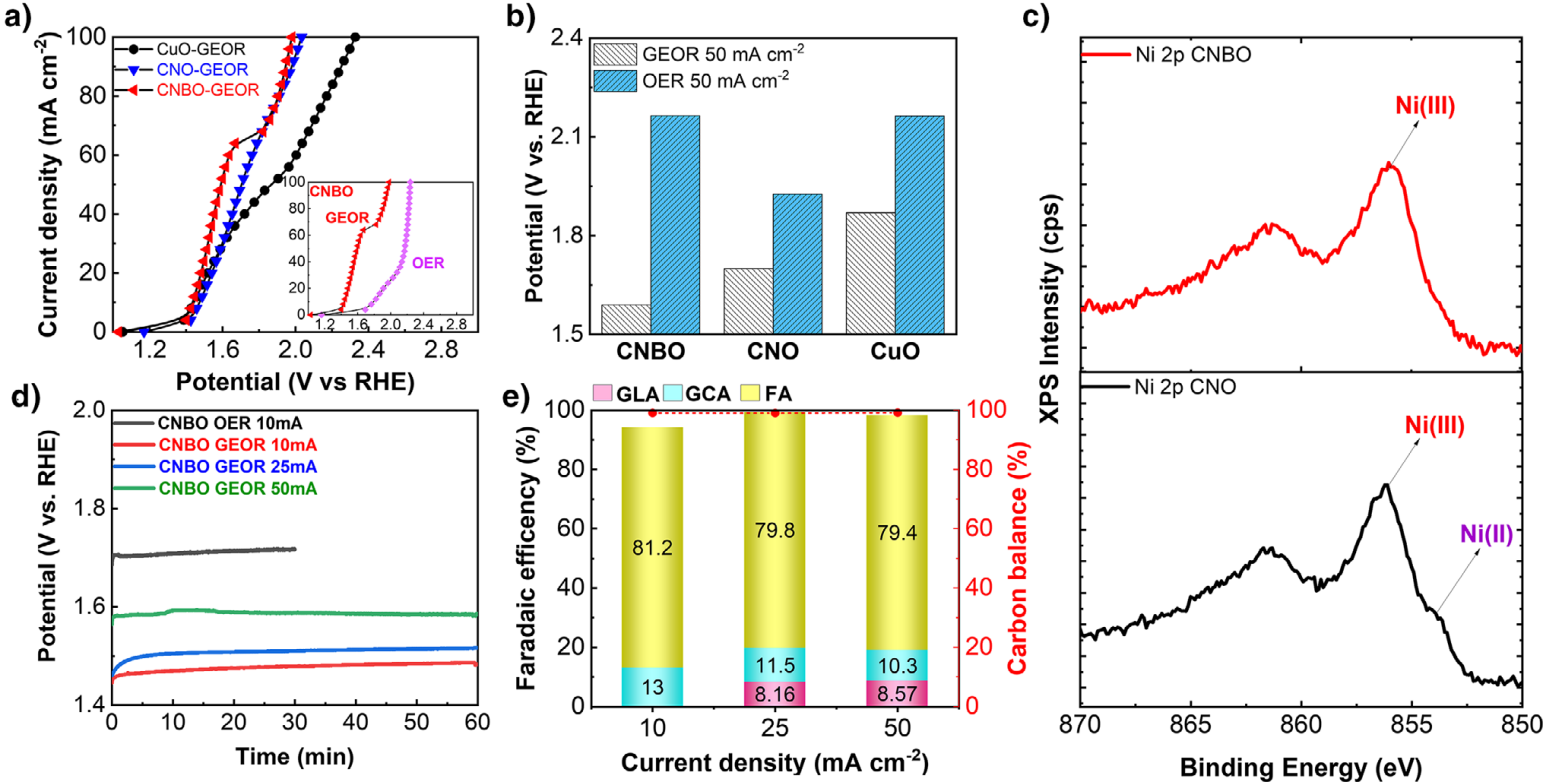
Electrochemical and surface characterization of GEOR electrocatalysts. a) Galvanostatic linear sweep voltammetric traces collected on CNBO and control samples and b) retrieved potentials required to achieve a current density of 50 mA cm^−2^. c) High‐resolution Ni 2*p_3/2_* XPS spectra collected on CNO and CNBO. d) Chronopotentiometric scans and e) related product distribution obtained over CNBO at different current densities. GLA: glyceric acid, GCA: glycolic acid, FA: formic acid.

At first, all the samples were preconditioned by cyclic voltammetry (CV). Insights on the electrocatalytic activity of the samples could be retrieved from their CV traces (Figure S9 and related discussion). Particularly striking is the major difference in the onset potentials of OER (≈1.68 V vs. RHE, measured in 1 M KOH) and GEOR (≈1.40 V vs. RHE, measured in 1 M KOH + 0.1 M glycerol) over CNBO (and, partially, CNO), which is consistent with the thermodynamic considerations discussed in the introduction. From a practical point of view, this winning competition of GEOR over OER on CNBO offers a large potential window in which no parasitic OER is expected. Figure 2a reports the galvanostatic linear sweep voltammetry (GLSV) collected with CNBO, CNO, and CuO, without IR compensation (Figure S10 and related discussion). In accordance with CVs, both CNBO, CNO, and CuO showed similar onset potentials for GEOR, around 1.40 V vs. RHE, which is noticeably less anodic than the onset potential for OER on the same materials (inset in Figure 2a). The electrochemical performance toward GEOR and OER activity for CNBO and other control samples was evaluated by undertaking long‐term galvanostatic measurements at an applied current density of 50 mA cm^−2^. As shown in Figure 2b, among all, CNBO required the lowest and highest bias for GEOR (1.58 V) and OER (2.23 V), respectively, thus outperforming CNO and CuO in terms of both GEOR activity and OER suppression ability. This further highlights the importance of the incorporation of Bi and its role in the selective oxidation of glycerol over OER. Figure 2c reports the high‐resolution Ni 2*p* XPS spectra of CNO and CNBO. Apart from the satellite peaks in the binding energy region from 860 to 870 eV, the Ni 2*p_3/2_* spectrum of CNO evidenced a main peak centered at approx. 856 eV, and a lower intensity one at approx. 853.6 eV, assigned to Ni^3+^ and Ni^2+^ species, respectively.^[^ ^42^ ^]^ However, when we moved to CNBO (i.e., in the presence of Bi), the Ni 2*p_3/2_* spectra showed solely the peak assigned to Ni^3+^. Compared to Ni^2+^ ones, Ni^3+^ species have been demonstrated to be more active in forming nickel oxyhydroxide (NiOOH)—the actual active site for oxidations in Ni‐based catalysts—since the adsorption energy of OH is lower on Ni^3+^ than on Ni^2+^ (−0.98 eV instead of −0.75 eV).^[^ ^43^ ^]^ This modification of the electronic structure of nickel active sites, enabled by bismuth, contributes to the faster kinetics registered for CNBO (e.g., the higher slope in the GLSV trace in Figure 2a), while the suppression of OER is consistent with previous reports.^[^ ^4^ ^]^ Thus, the introduction of Bi tuned the electronic density of the Ni sites and thus activity, leading to significant promotion of the electrocatalytic performance of CNBO.

The activity, stability, and GEOR selectivity of CNBO were then tested by means of 1‐h long chronopotentiometric (CP) scans at different current densities ranging from 10 to 50 mA cm^−2^ (Figure 2d). No obvious shift in the potential was detected, indicating the stability of the CNBO catalyst in this timescale and under these operative conditions. A comparison between 10 mA cm^−2^ CP scans in the presence and absence of glycerol (Figure 2d) further emphasized the anodic voltage reduction achievable when replacing OER with GEOR. Indeed, the required potential for sustaining the reaction rate with OER was 273 mV higher than that with GEOR.

The electrolytes from the galvanostatic studies using the CNBO catalyst at different applied current densities were then analyzed by high‐performance liquid chromatography (HPLC) to assess the GEOR product distribution. A comprehensive description of the analytical techniques used for GEOR (and CO_2_RR) products detection and quantification, and related calibration curves is available in the Supporting Information (Figures S11–S13). As evident from Figure 2e, formic acid (present in the alkaline electrolyte as formate) was the dominant product over all the investigated current range, with an FE of ca. 80%, while glycolic and glyceric acid were detected in lower amounts (8%–10%). It is also important to notice that both the overall FE and the carbon balance of the process approached unity, implying a complete GEOR selectivity over OER (additional details on O_2_ detection available in the Supporting Information, Figure S14, and related discussion) and an almost null production of CO_2_ (from the undesired deep oxidation of glycerol). Based on the nature of the GEOR products and on the results we obtained by their individual oxidation on CNBO (Figure S15a–c), we infer that glycerol oxidation to formic acid/formate proceeds through the following steps: glycerol→ glyceric acid → glycolic acid → formic acid (Figure S15d).

The electrochemical performance of CNO, CuO, and hollow CNBO particles (CuO core removed by leaching) was also evaluated under identical conditions, with the results presented in Figures S16–S18 and related discussion. Under galvanostatic measurements, CuO exhibited a stable performance similar to CNO and CNBO at an applied current density of 10 mA cm^−2^ (Figure S16a). However, at higher applied current densities of 25 and 50 mA cm^−2^, CuO exhibited significant initial decay, indicating its instability under these operating conditions (inset, Figure S16a). In contrast, although CNO exhibited stable electrochemical performance over the 1‐h electrolysis duration (Figure S16b) across the entire current range, its GEOR performance was significantly worse, showing higher GEOR overpotentials at specific current densities compared to CNBO (Figure S16c). Interestingly, the GEOR selectivity of CuO and CNO was also dominated by formic acid but the carbon balance and overall FE of the process indicate significant contributions from the carbon substrate oxidation and OER competition (Figure S16d,e and related discussion). Hollow CNBO particles also showed a worse GEOR performance when compared to the actual CNBO catalyst, with the absence of the Cu‐based core negatively impacting the charge transfer resistance of the catalytic system (Figure S18).

The stability of the materials was then evaluated by means of structural, morphological, compositional, surface, and electrochemical characterization carried out on post‐electrolysis electrodes. CNBO retained better electrochemical performance and chemical–physical features in comparison to CuO and CNO, as evident from GLSV curves, SEM imaging, and Raman spectroscopy (Figures S19–S22). Notably, CNBO retained its typical core‐shell‐like structure (STEM‐EDS analysis, Figure S22). The only altered feature of CNBO after GEOR testing was its surface composition (XPS, Figure S23). Consistent with the observations made for used CuO electrodes (Figure S16d and TableS2), the concentration of Cu decreased, resulting in a significant increase of Ni content on the surface of the particles.

The durability of CNBO has also been tested. At first, we assessed the long‐term stability of the catalyst by performing a 20 h‐long electrolysis test at 50 mA cm^−2^ under the above‐described H‐cell configuration (Figure S24). Over the first 10 h, the FE_GEOR_ remained above 90%, with the carbon balance reaching unity (Table S3). A slight positive shift in potential was observed due to the consumption of glycerol, which totally reversed upon electrolyte replacement after 10 h of reaction. Overall, CNBO exhibited good stability and durability, as demonstrated by the consistent polarization profile after 20 h of operation (Figure S25).

**Figure 3:**
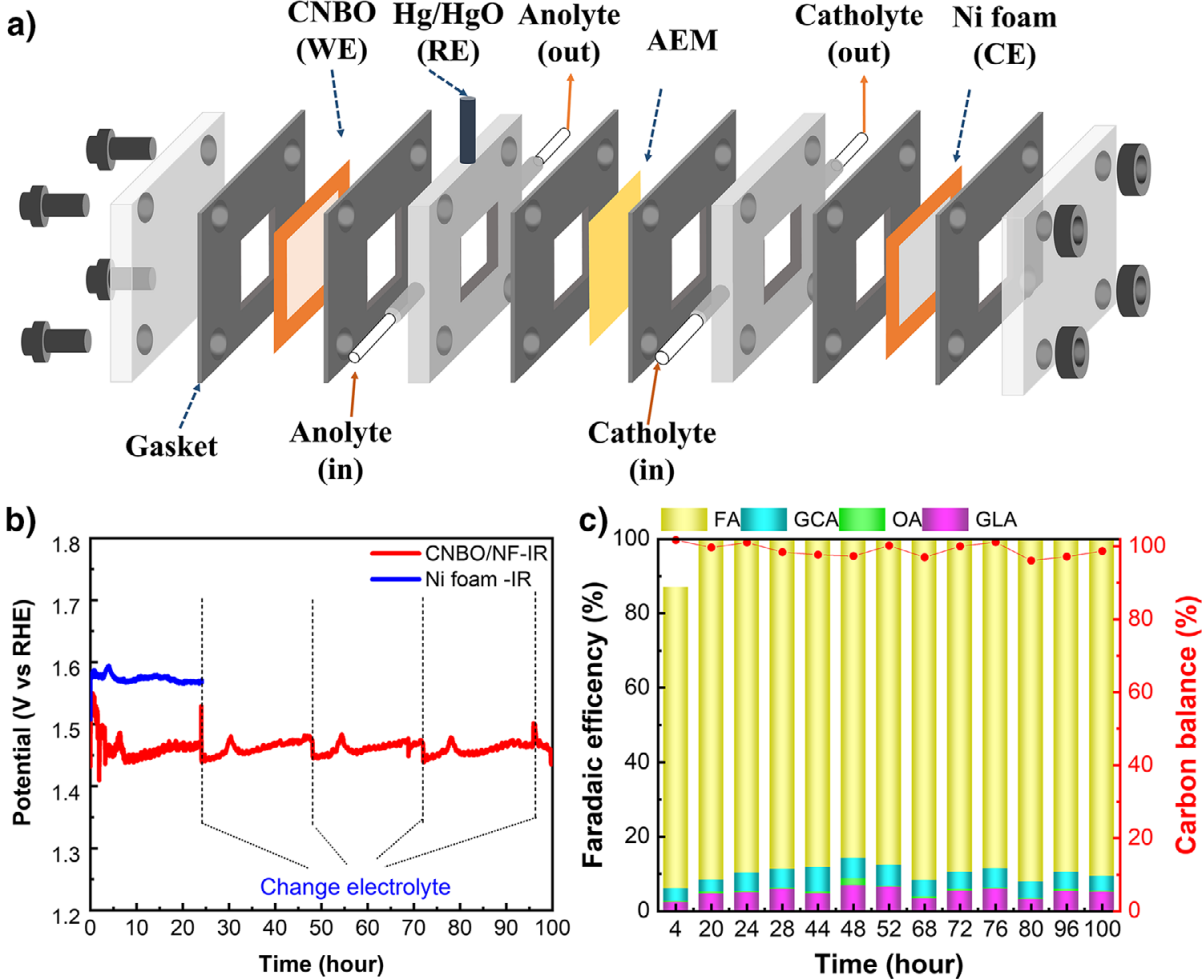
a) Exploded view of the flow cell used in 100‐h stability test, b) Chronopotentiometric scans, recorded at 100 mA cm^−2^ over CNBO/NF and NF with IR correction, c) related product distribution obtained over CNBO/NF. GLA: glyceric acid, GCA: glycolic acid, OA: oxalic acid, FA: formic acid.

With the purpose of further assessing the stability and electrochemical performance of CNBO, this time under more industrially relevant conditions, we opted for a longer test of 100 h under a different cell configuration, namely, a two‐compartment three‐electrode flow cell (Figure 3a). After having optimized the electrode fabrication (i.e., implementing metallic substrates in place of less stable carbon‐based ones) and the overall cell configuration, we have been able to achieve stable electrochemical performance. An IR‐corrected anodic potential of ca. 1.47 V versus RHE has been recorded at 100 mA cm^−2^, complemented by a stable product distribution yielding an almost unitary FE_GEOR_ and a constant FE to formic acid/formate of *ca*. 90% (Figure 3c). Additional electrochemical data and the characterization of the post‐electrolysis electrodes, further corroborating the remarkable stability of the material are available in the Supporting Information (Figures S26d–S27 and related discussion).

To position our work within the field, we compared our results to some leading studies using Ni‐based catalysts for GEOR (Table S4). Although it is challenging to properly normalize the current density delivered at a specific potential because of the variety of substrates and mass loadings used in different studies, CNBO ranks among the best electrocatalysts in terms of FE_GEOR_.

### In situ Raman Spectroscopic Studies

**Figure 4:**
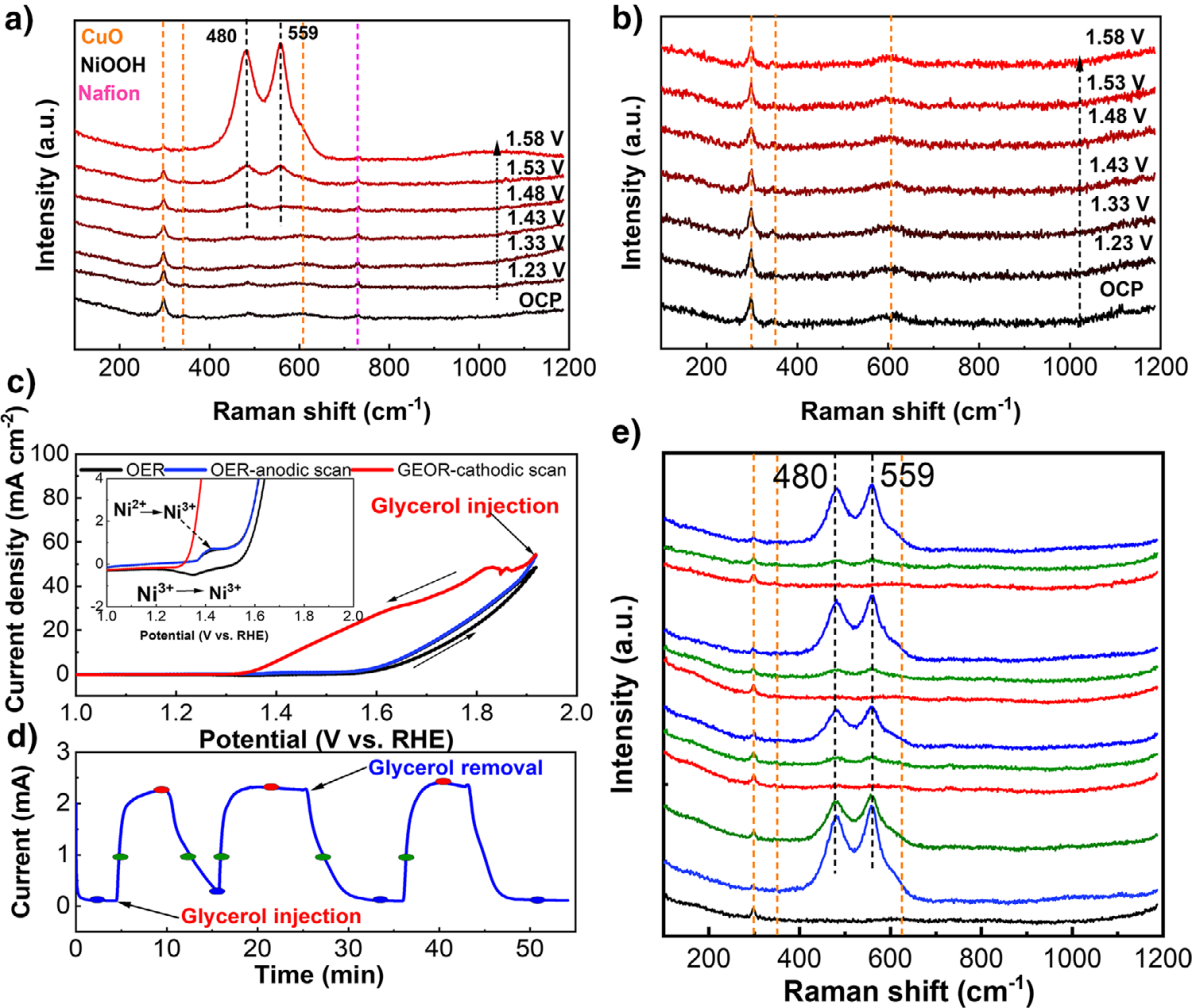
In situ Raman spectra collected on CNBO at different potentials under a) OER and b) GEOR reaction conditions. c) CV scans collected on CNBO under OER conditions and after glycerol injection, scan rate = 1 mV s^−1^. d) I‐t trace, recorded at 1.53 V versus RHE, of interleaved OER and GEOR, and e) related in situ Raman spectra registered at different time.

With the aim of exploring the structural and compositional evolution of the catalyst surface under operative conditions, we performed in situ Raman experiments. The setup used is depicted in Figure S28 and described in the related paragraph. At first, we focused on monitoring the surface evolution of CNBO under OER conditions (i.e., 1 M KOH electrolyte) and at different applied potentials, ranging from 1.23 to 1.58 V versus RHE. Due to the relatively weak intensity of nickel and bismuth oxides Raman peaks, no signals were detected in the potential window from open circuit potential (OCP) to 1.43 V versus RHE with the exception of the peaks assigned to CuO (Figure 4a). Two new Raman peaks at 478 and 550 cm^−1^ appeared when the applied potential exceeded 1.48 V versus RHE. These peaks correspond to the e_g_ bending and A_1_ _g_ stretching vibration mode of Ni^3+^‐O in Ni^III^‐OOH, proving the transformation of Ni^2+^ to NiOOH.^[^ ^20^, ^44^, ^45^ ^]^ Although 1.43 V versus RHE is the onset potential for the oxidation of Ni^2+^ to Ni^3+^ (according to CV measurement, Figure S9), the amount of NiOOH formed at this potential appears to be too small for detection. Along with the potential increase, the intensity of the NiOOH peaks increased, indicating that NiOOH became the dominant species on the catalyst surface and served as the active site for OER, as largely agreed in the literature.^[^ ^44^ ^]^ In contrast, under GEOR conditions (i.e., 1 M KOH + 0.1 M glycerol electrolyte), the CNBO showed no obvious changes across the potential range of 1.23–1.58 V (Figure 4b). Therefore, either NiOOH did not form under GEOR conditions, or the presence of glycerol caused a fast reduction of Ni(III) species, basically acting as a scavenger. Aiming at shedding light on the CNBO surface evolution in the presence of glycerol, we designed an ad hoc experiment.

Briefly, a CV scan was run in 1 M KOH on a CNBO electrode, thus forming NiOOH on the surface. Glycerol was injected at the end of the anodic scan, right before the scanning direction was inverted (Figure 4c). As a consequence, the Ni(III) reduction peak (1.38 V versus RHE) disappeared, suggesting that NiOOH species had been consumed by glycerol and supporting the assumption of an indirect oxidation mechanism where NiOOH acts as a mediator. To confirm this hypothesis, in situ Raman spectra were recorded in alternating OER and GEOR conditions. In detail, we used the same in situ Raman setup but periodically changed the electrolyte composition: from 1 M KOH (OER) to 1 M KOH + 0.1 M glycerol (GEOR). Meanwhile, a constant potential was applied to the working electrode. OER and GEOR took turns over three cycles at 1.53 V versus RHE (Figure 4d). Raman spectra were recorded at different stages, as indicated by the marked points in the I‐t trace. In Figure 4e, Raman spectra consistently show the formation of NiOOH species during OER (blue spectra). Upon glycerol feeding, the intensity of the NiOOH peaks decreased (green spectra), and ultimately disappeared completely (red spectra) once the GEOR electrolyte fully replaced the OER electrolyte. These trends reversed upon switching back to the OER electrolyte, proving the reversibility of the phenomenon. Our observations are consistent with an indirect mechanism for glycerol oxidation, in which NiOOH, continuously formed upon anodic bias, acts as a mediator for glycerol oxidation.^[^ ^11^, ^46^ ^]^ The impossibility of detecting the typical NiOOH Raman peaks can be attributed to the fast reaction rate between (oxy)hydroxides and adsorbed glycerol.^[^ ^47^, ^48^ ^]^ As a result, NiOOH did not accumulate enough to be detected by Raman during GEOR. Based on these observations, we can conclude that NiOOH species formation is a crucial step in the reaction mechanism and that electrocatalysts able to foster their generation at lower applied potentials are expected to be more efficient.

### Full Cell Studies: Coupling GEOR with CO_2_RR

After evaluating the performance and stability of CNBO and having shed light on the GEOR mechanism, we moved on to investigate the possible coupling of GEOR (in replacement of OER) with CO_2_RR under industrially relevant conditions, i.e., in an electrochemical flow cell implementing a gas diffusion electrode (GDE) configuration. Experimental details on the flow cell setup are available in the Supporting Information (Figure S29 and related paragraph).

Considering the high selectivity of CNBO toward the production of formic acid from glycerol, we designed two different full‐cell configurations. The first implemented a silver‐based cathode (Ag/PTFE) for CO‐selective CO_2_RR, while the second used a tin‐based cathode (Sn/Sigracet 39BB) for formic acid‐selective CO_2_RR. The rationale behind these choices stems from the ease of product separation and single‐product throughput, respectively.

**Figure 5:**
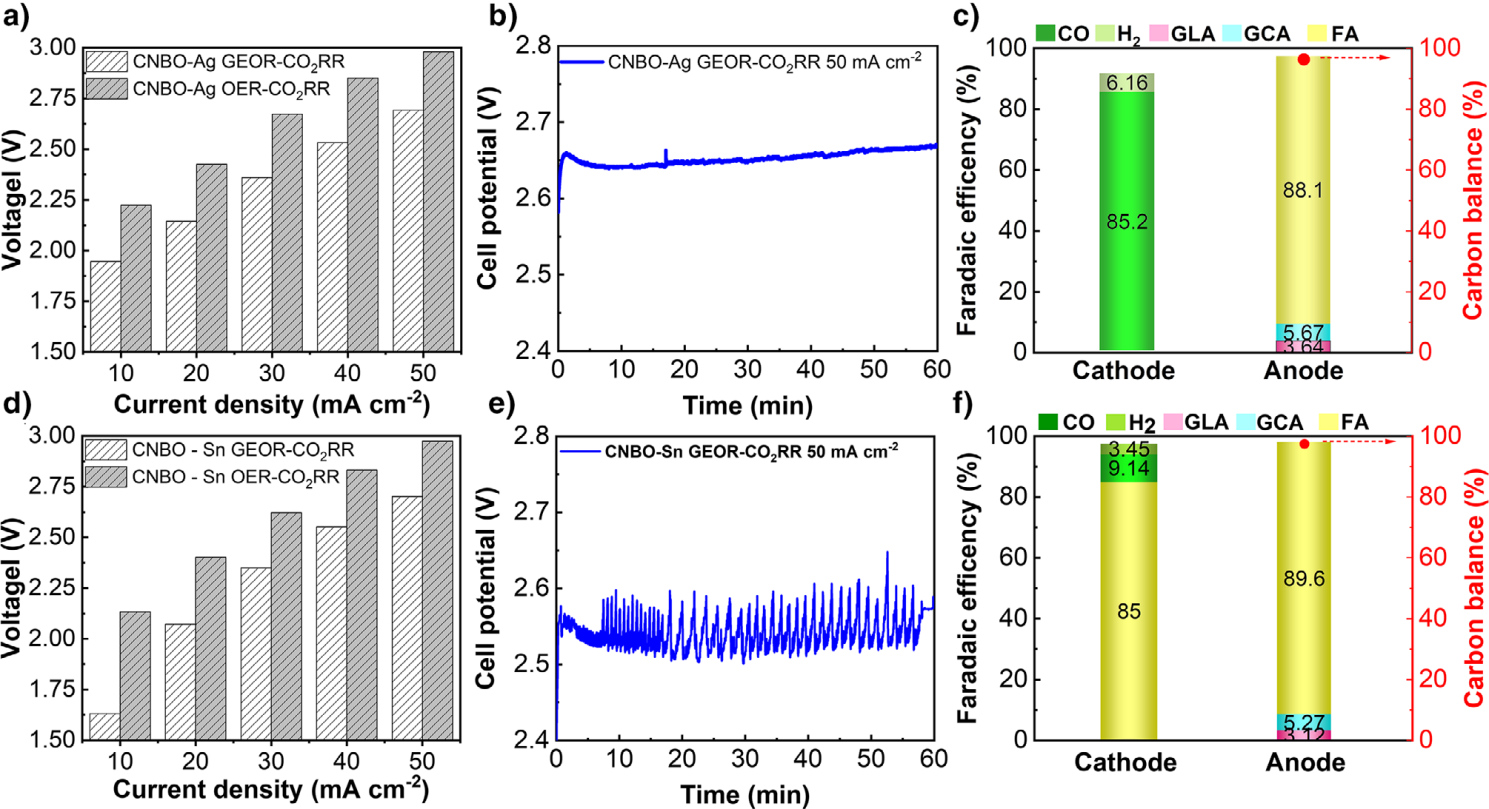
Cell voltage required to achieve specific current densities in different full cells: a) CNBO‐Ag/PTFE, d) CNBO‐Sn/ Sigracet 39BB. CP scans collected at 50 mA cm^−2^ implementing b) CNBO + Ag/PTFE, e) CNBO + Sn/Sigracet 39BB configurations, c,f) and related product distribution.

The electrochemical features of the two systems were examined by GLSV and GEIS (Figure S30, Supporting Information). In both cases, replacing OER with GEOR on CNBO resulted in a lower cell voltage, thus improving energy consumption. Indeed, all current density within the range of 10–50 mA cm^−2^ could be achieved at the cell voltage of ≈ 300 mV lower when GEOR is implemented (Figure 5a,d, and Figure S30b,c). At the same reaction rate, the operational cost could be decreased by 10%–25% with the current density range from 10 to 50 mA cm^−2^ when GEOR replaces OER (Figure S30d,e and detailed calculation in Supplementary Note1). Moving to chronopotentiometric scans implementing GEOR at the anode, the full cell assembled with CNBO and Ag/PTFE electrodes delivered 50 mA cm^−2^ at the overall cell potential of ca. 2.65 V (Figure 5b). Consistently with H‐cell testing, anodic mass, and charge balances reached near‐unity values. Interestingly, the FE towards formic acid increased (≈ 88% versus ≈ 80% obtained from H‐cell testing), while glycolic and glyceric acid FEs halved (Figure 5c). At the cathode, the total FE reached 91% with FE_CO_ ≈ 85.2% and traces of hydrogen (FE_H2_ ≈ 6.2%).

Implementing the CNBO + Sn/Sigracet 39BB configuration and applying the same operational condition, the full cell voltage was around 2.55 V (Figure 5e). The CP trace obtained under this configuration is noisy; we tentatively assigned this behavior to the progressive flooding of the cathode. The CNBO anode retained its GEOR selectivity towards formic acid (FE_FA_ ≈ 89.6%), while the Sn‐based cathode yielded a FE_FA_ ≈ 85%. Other CO_2_RR products were detected in lower amounts (FE_CO_ ≈ 9.14% and FE_H2_ ≈ 3.45%) (Figure 5f).

In addition to the reduction in the operational cost of the electrolysis, we also considered the energy consumption needed for the generation of products by CO_2_RR and GEOR. We calculated the specific production energy for formic acid production (J mol^−1^_FA_) in a GEOR‐CO_2_RR electrolyzer, demonstrating a 52.5% reduction compared to the values obtained in a traditional OER‐CO_2_RR electrolyzer (detailed calculations are provided in Supplementary Note2). This result stems from (i) the lower operating voltage, due to GEOR‐based anode depolarization and (ii) the simultaneous production of formic acid in both cell compartments. Indeed, the same electrons contribute to formic acid generation at both electrodes, with electrons used to produce formic acid by GEOR at the anode traveling to the cathode to reduce CO_2_. Therefore, this study introduces single‐product co‐generation as an effective tool to halve the specific production energy of formic acid.

Finally, compared to previous articles proposing OER alternatives for CO_2_ electrolysis, we note that our work is competitive in terms of full‐cell voltage operation and selectivity toward high added‐value products (Table S6). Importantly, the design of the flow cell used in this study is not optimized, resulting in high system impedance due to the long distance between the anode and cathode (Figure S30f–h). Thus, supported by a proper engineering of the cell configuration and geometry (e.g., reducing ohmic losses by moving to a zero‐gap configuration), the metrics here reported can be further improved, making OER alternatives like GEOR increasingly interesting in the landscape of electrosynthetic technologies.

## Conclusion

In conclusion, we have demonstrated that CNBO exhibits good GEOR activity, characterized by complete OER suppression while achieving FE_GEOR_ ≈ 100% at the high current of 100 mA cm^−2^ for 100‐h electrolysis. By correlating XPS data and electrochemical characterizations of CNBO, we revealed that Bi insertion into the catalyst matrix improved catalytic performance by tuning the electron density of the Ni active sites. A combination of electrochemistry and in situ Raman spectroscopy studies confirmed the formation of NiOOH under oxidation bias, which were identified as the active sites for GEOR. In the presence of glycerol, NiOOH was rapidly consumed, supporting the GEOR indirect oxidation mechanism. Finally, the coupling of GEOR with CO_2_RR was demonstrated to lower operational electrolysis cost up to 25%, while full‐cell configurations designed for the co‐generation of a single product are introduced as a promising tool to theoretically halve the specific energy production of electrosynthetic fuels and/or chemicals.

## Conflict of Interests

The authors declare no conflict of interest.

## Supporting information



Supporting Information

## Data Availability

The data that support the findings of this study are available from the corresponding author upon reasonable request.
