# Supplementary material for: Coupling of CuO@NiBiOx Catalyzed Glycerol Oxidation to Carbon Dioxide Reduction Reaction for Enhanced Energy Efficiency
Source: Angew Chem Int Ed Engl. 2025 Apr 17;64(25):e202502617. doi: 10.1002/anie.202502617 (PMC12171342; doi:10.1002/anie.202502617)
Supplement: Supplementary file 1 — Supporting Information [file ANIE-64-e202502617-s001.docx]

**Supporting Information to**

**Coupling of CuO@NiBiO_x_ Catalyzed Glycerol Oxidation to Carbon Dioxide Reduction Reaction for Enhanced Energy Efficiency**

Thi-Hong-Hanh Le^1,2^, Yong Zuo^1,3^, Manjunath Chatti^1^, Martina Rizzo^1,4^, Andrea Griesi^1^, Abinaya Annamalai^1^, Simone Lauciello^1^, Luca Leoncino^1^, Mirko Prato^1^, Silvia Dante^1^, Ilka Kriegel^1,4^, Giorgio Divitini^1^, Michele Ferri^1^*, Liberato Manna^1^*.

1. Istituto Italiano di Tecnologia (IIT), Via Morego 30, Genova, Italy
2. Università degli studi di Genova (UniGe), Via Dodecaneso 31, Genova, Italy
3. School of Chemistry and Chemical Engineering, Chongqing University, Chongqing, 400044, China
4. Dipartimento di Scienze Applicate e tecnologia (DISAT), Politecnico di Torino, Corso Duca degli Abruzzi 34, Torino, Italy

Contents

[Chemicals and Materials 3](#_Toc194270445)

[Chemicals for Cu_2_O synthesis 3](#_Toc194270446)

[Chemicals for CuO@NiBiO_x_ and CuO@NiO_x_ synthesis 3](#_Toc194270447)

[Chemicals for glycerol oxidation 3](#_Toc194270448)

[Chemicals and materials for electrode preparation and electrochemical experiments 4](#_Toc194270449)

[Material characterization 4](#_Toc194270450)

[Electrode fabrication, electrochemical setup, and testing 5](#_Toc194270451)

[Material synthesis 7](#_Toc194270452)

[Synthesis of Cu_2_O core 7](#_Toc194270453)

[CuO@NiO_x_, CuO@NiBiO_x_ core-shell-like preparation 9](#_Toc194270454)

[Optimization of the CNBO synthetic route 9](#_Toc194270455)

[Optimized CNBO synthesis and preliminary characterization 11](#_Toc194270456)

[Surface composition of samples (XPS analyses) 15](#_Toc194270457)

[Electrochemical testing in H-cell – Additional details 16](#_Toc194270458)

[Figure S10. Nyquist plots (a) and fitted R_1_ data (b) of CuO, CNO and CNBO under GEOR condition at the current of 2.5 mA. 19](#_Toc194270459)

[GEOR and CO_2_RR products analysis 19](#_Toc194270460)

[GEOR products analysis 19](#_Toc194270461)

[CO_2_RR products analysis 22](#_Toc194270462)

[Checking OER activity over CNBO - Gas chromatography 23](#_Toc194270463)

[Reaction pathway 24](#_Toc194270464)

[Electrochemical performance of control materials 25](#_Toc194270465)

[Post-electrolysis analyses – Catalysts stability 30](#_Toc194270466)

[*In-situ* Raman spectroscopy analyses – Catalyst evolution under OER and GEOR conditions 40](#_Toc194270467)

[GEOR + CO_2_RR coupling – Additional data 41](#_Toc194270468)

[Supplementary Notes 43](#_Toc194270469)

[Supplementary Note 1 43](#_Toc194270470)

[Supplementary Note 2 43](#_Toc194270471)

[Reference 47](#_Toc194270472)

# Chemicals and Materials

## Chemicals for Cu_2_O synthesis

Copper(II) chloride dihydrate (CuCl_2_.2H_2_O, 99.99% purity, purchased from Sigma Aldrich), L-Ascorbic acid (AA, 98% purity, purchased from Sigma Aldrich), ammonia solution (NH_3_.H_2_O, 25% v/v%, purchased from Fisher Scientific), Poly(ethylene glycol)-block-poly(propylene glycol)-block-poly(ethylene glycol) ((C_3_H_6_O.C_2_H_4_O)_x_, P123 MW 2,900, purchased from Sigma Aldrich).

## Chemicals for CuO@NiBiO_x_ and CuO@NiO_x_ synthesis

Nickel (II) chloride (NiCl_2_, 99.9% purity, purchased from Sigma Aldrich), bismuth (III) nitrate pentahydrate (Bi(NO_3_)_3_.5H_2_O, 98% purity, purchased from Sigma Aldrich), sodium thiosulfate (Na_2_S_2_O_3_, 99% purchased from Sigma Aldrich), polyvinylpyrrolidone, average MW 40,000 ((C_6_H_9_NO)_x_ PVP40T, purchased from Sigma Aldrich).

## Chemicals for glycerol oxidation

Glycerol (GLY, 99.5% purity, purchased from Sigma Aldrich), glyceraldehyde (GLAD, > 90% purity, purchased from Sigma Aldrich), dihydroxyacetone (DHA, 98% purity, purchased from Sigma Aldrich), glyceric acid (GLA, 20% in water ≈ 2 M, purchased from Tokyo Chemical Industry-TCI), glycolic acid (GCA, 99% purity, purchased from Sigma Aldrich), formic acid (FA, 98% wt.% purity, purchased from TCI), tartronic acid (TA, 97% purity, purchased from Fisher Scientific), lactic acid (LA, 85% purity, purchased from Sigma Aldrich), oxalic acid (OA, 99% purity, purchased from Sigma Aldrich).

## Chemicals and materials for electrode preparation and electrochemical experiments

Carbon paper (AvCarb MGL 370, purchased from FuelCellStore), sulfuric acid (H_2_SO_4_, 95-97% purchased from Sigma Aldrich), nitric acid (HNO_3_, 65% wt.%, purchased from Sigma Aldrich), potassium hydroxide (KOH, 85% purity, purchased from Sigma Aldrich), isopropanol (IPA, ≥99.8% purity, purchased from Sigma Aldrich), ethanol (EtOH, 99.8% purity, purchased from Sigma Aldrich), Chelex 100 resin (Bio-Rad, catalog no. 210011676).

All aqueous solutions have been prepared using MilliQ water (18.2 Ώ, Millipore). All chemicals were used without further purification.

# Material characterization

**Scanning Electron Microscopy (SEM**). The samples were examined on a Zeiss Gemini SEM 560 Field Emission SEM (FE-SEM). **Energy Dispersive X-ray Spectroscopy** **(EDS)** analyses were performed using a detector X-Max (80 mm^2^ area, Silicon Drift Detector, Oxford Instruments). The mapping of Cu and O was obtained by integration of the Cu L and O K peaks. **X-ray diffraction (XRD)** measurements were performed with a PANalytical Empyrean using Cu K_α_ radiation. **X-ray photoelectron spectroscopy** **(XPS)** measurements were conducted with a Kratos Axis UltraDLD spectrometer using a monochromatic Al K_α_ source, operated at 20 mA and 15 kV. High-resolution spectra were acquired using a pass energy of 10 eV. Spectra were calibrated based on the mainline of the carbon 1 s spectrum set to 284.8 eV. **Raman** spectra were acquired on an inVia Renishaw Raman microscope using an excitation HeNe laser of 633 nm and a nominal power of 17 mW. The spectrometer was equipped with a back illuminated CCD detector and with an 1800 l/mm diffraction grating, giving a spectral resolution better than 1 cm^-1^. All the measurements were conducted in backscattering geometry at room temperature. Prior to experiments, the Raman spectrometer was calibrated with a silicon reference to (520.5 ± 0.2) cm^−1^. For all measurements, the software WiRE 5.5 was used. The samples were focused by a 50× long working distance objective providing a spot size of about 1 μm in diameter. As for the further electrochemical test, we coated the prepared powder materials on carbon paper with Nafion binder. The coated electrodes were utilized for characterizing by Raman. Carbon paper substrate, carbon paper coated with Nafion, and commercial Bi_2_O_3_ were also checked to provide the reference peaks.

**Scanning** **transmission electron microscopy** **(STEM).** Preliminary sample characterizations were performed on JEM1400Plus (JEOL) operated at 120kV, equipped with LaB_6_ thermionic source and with JED-2300 EDS silicon drift type detector (JEOL). Further material images were acquired on a probe corrected Thermo Fisher Spectra 300 S/TEM operated at 300 kV. The EDS signal was collected by a Dual-X system comprising two detectors, one on either side of the sample, for a total acquisition solid angle of 1.76 sr. Compositional maps were acquired using rapid raster scanning in Velox, with a probe current of ∼230 pA. Elemental maps were produced within Velox. NC solutions were diluted 5 times in Ethanol and then drop-cast onto gold TEM grids with an ultrathin carbon film. The line EDS was obtained averaging 360-line profiles collected from the particle center to the particle surface. Data processing was performed by the means of Hyperspy, an open source Pyhton library written for the analysis of multidimensional datasets such as STEM-EDS data^[1]^ .

# Electrode fabrication, electrochemical setup, and testing

Catalytic inks were made from catalysts dry powders by dispersing the synthesized materials in isopropanol at the concentration of 2.5 mg/mL. Nafion was used as a binder with a 40 wt.% ratio with catalysts. The mixture was sonicated for 30 minutes as to ensure inks homogeneity. The Av Carb MGL 370 carbon paper, which served as the substrate for all samples, was pretreated using a concentrated solution of sulfuric acid (H_2_SO_4_) and nitric acid (HNO_3_) in a 3:1 volume ratio. The treatment was carried out for 15 minutes. This pretreatment step effectively removed contaminants from the carbon paper and enhanced its hydrophilic properties, making it more suitable for further electrochemical tests. The electrode was crafted by drop casting a known volume of the catalyst ink as to obtain a final loading of 0.125 mg catalyst on each 0.25 cm^2^ treated carbon paper. KOH aqueous solution, used as electrolyte (either with the addition of glycerol or not), has been treated with Chelex 100 resin (Bio-Rad, catalog no. 210011676) before use to remove common metal-based contaminants^[2]^.

The catalytic performance of the catalysts was evaluated by Cyclic Voltammetry (CV), Galvanostatic Linear Sweep Voltammetry (GLSV), and Chronopotentiometry (CP). The electrolyte for blank (*i.e.*, OER) tests was 1 M KOH, while the electrolyte for the actual GEOR testing consisted of 1 M KOH + 0.1 M glycerol. The impedance spectrum of the system was investigated by Galvanostatic Electrochemical Impedance Spectroscopy (GEIS), conducted at 2.5 mA in a frequency range from 100 kHz to 0.05 Hz with 0.5 mA amplitude. All the reported potentials were converted from the reference scale (Hg/HgO) to the reversible hydrogen electrode (RHE) scale by applying the equation:

*E_RHE_ = E^0^_HgO/Hg_ + E_HgO/Hg_ +0.059*pH - IR_u_.*

where E^0^_HgO/Hg_ has been retrieved from frequent calibrations of the reference electrode versus a standard RHE (HydroFlex, purchased from Gaskatel), and the uncompensated system resistance (R_u_) was determined by GEIS.

Details on the cell and electrodes configuration are available here in the Supporting Information, at sections dedicated to H-cell and flow cell testing of catalysts.

# Material synthesis

## Synthesis of Cu_2_O core

Copper (I) oxide spheres were prepared by reducing CuCl_2_ with ascorbic acid. The procedure is depicted in **Figure S1**. Accordingly, 1.836 g of P123 were dissolved in 180 mL Milli-Q water at 291 K under constant stirring (800 rpm) for 3 h. The dark blue complex of Cu(NH_3_)_4_^2+^ was prepared by adding 1.089 mL of an ammonia solution to 6.21 mL of a 0.2 M copper chloride dihydrate aqueous solution. Then, 6.75 mL of the prepared solution was added to the solution of surfactant under stirring. 15.0 mL of an ascorbic acid (0.6 M) solution was then injected by means of a syringe pump into the mixture at the rate of 0.78 ml/min. The solution was stirred for an additional 10 minutes, after which the resulting bright yellow precipitate was collected by centrifugation at 12,000 rpm for 10 minutes. The precipitate was washed several times with ethanol (as to remove any remaining polymer) and then dried under a nitrogen flow.


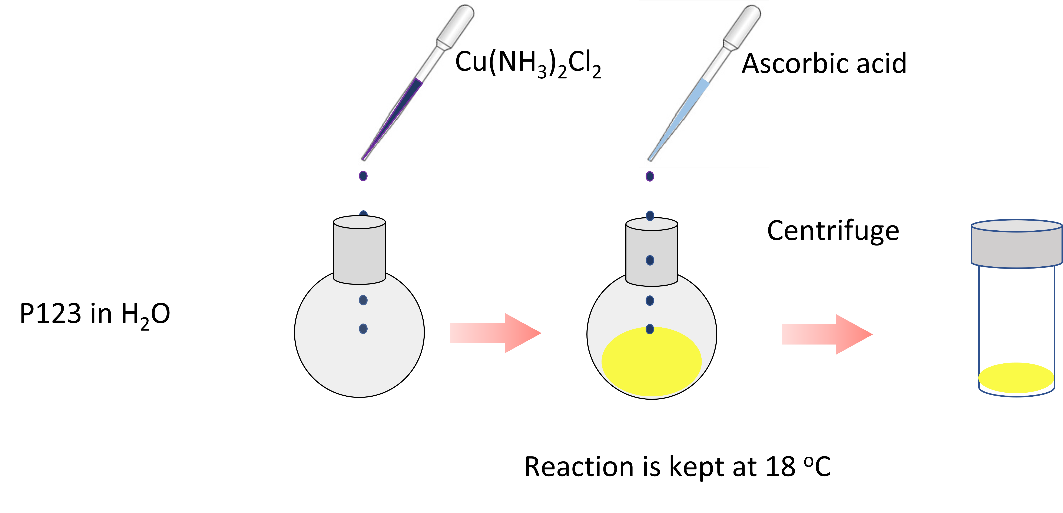


**Figure S1.** Graphical depiction of the Cu_2_O spheres synthetic procedure.

The desired spherical morphology of Cu_2_O was assessed by both SEM and TEM imaging (**Figure S2a,b**). Cu_2_O spheres display a large size distribution, with a diameter generally comprised between 150 and 400 nm, classifying them as microspheres. The homogeneous distribution of Cu and O throughout the particles was observed by SEM-Energy Dispersive X-ray Spectroscopy (SEM-EDS) (**Figure S2c**)**.** A confirmation of the phase and chemical composition of Cu_2_O spheres was obtained from X-ray diffraction **(**XRD) and Raman spectroscopy**.** As shown in the diffractogram reported in **Figure S2d**, all the diffraction peaks at 29.6, 36.4, 42.3, 61.4, and 73.5° (2θ°) match the standard reflections of Cu_2_O (JCPDS 96-900-5770). For what concerns the Raman spectrum, the detected peaks (140, 213, 290, 415, and 623 cm^-1^) were assigned to the T_1u_, 2_Eu_, A_2u_, 3 Γ_12_^−^ + Γ_25_^−^, and T_1u_ vibration modes of Cu_2_O ^[3]^. The small peaks at 290, 333, and 621 cm^-1^ represented the A_g_, B_g_ and B_g_ vibration modes of CuO ^[4]^ (**Figure S2e**), and their presence can be ascribed to the exposure of Cu_2_O to atmospheric oxygen (i.e., Cu(I) oxidation to Cu(II)).


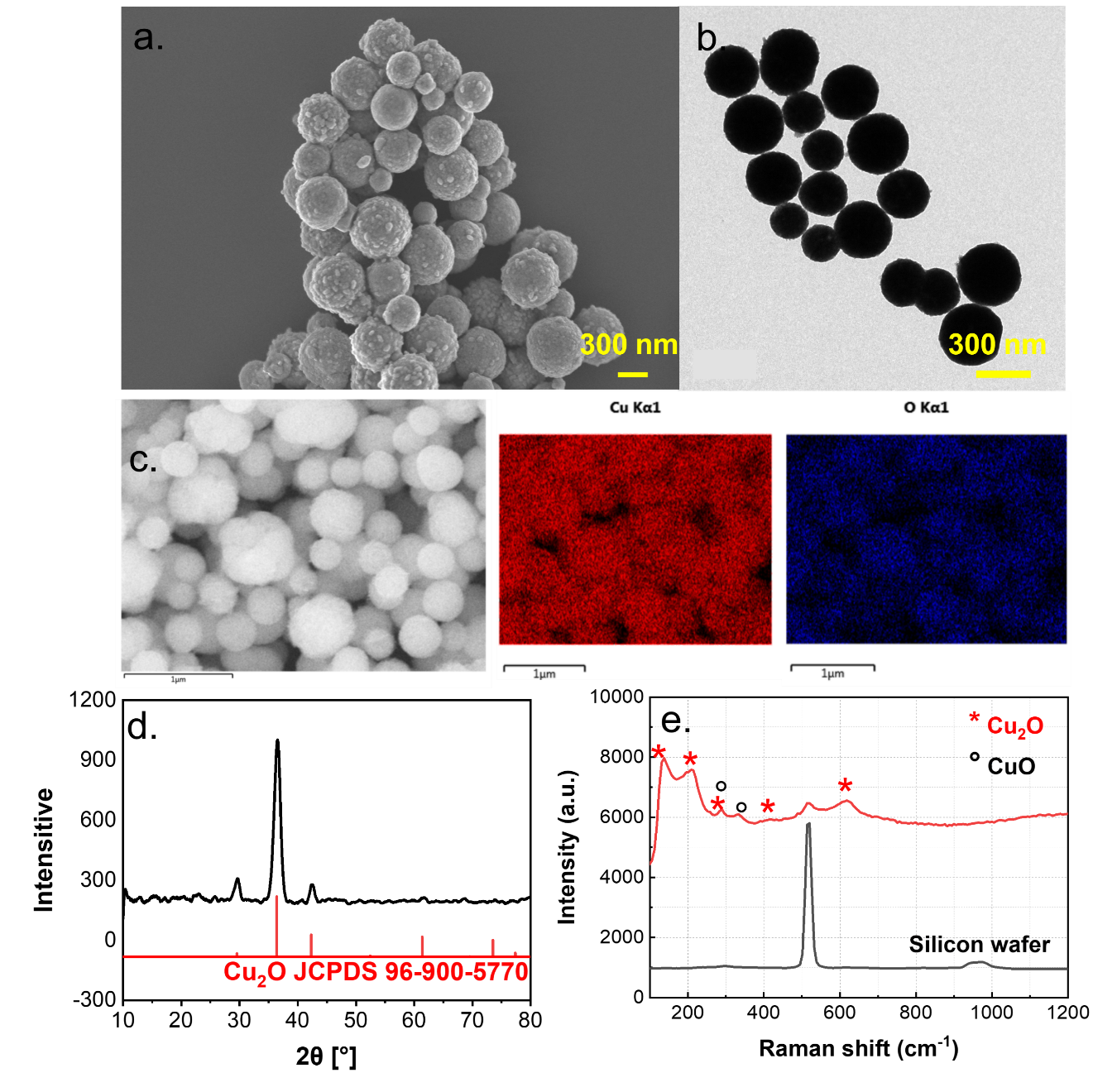


**Figure S2.** Full characterization of Cu_2_O spheres. (**a**) SEM and (**b**) Bright field-TEM images; (**c**) SEM-EDS mapping of Cu and O with the scale bars equal to 1µm; (**d**) XRD pattern and (**e**) Raman spectra.

## CuO@NiO_x_, CuO@NiBiO_x_ core-shell-like preparation

As described in the main text, CuO@NiO_x_ (CNO) and CuO@NiBiO_x_ (CNBO) core-shell-like catalysts were synthesized following a “Coordinating Etching and Precipitating” (CEP) method^[5]^ with some modification.

In detail, 60 mg of Cu_2_O spheres, (acting as both Cu source and hard templating agent), 7.8 mg of NiCl_2_ and 7.8 mg of Bi(NO_3_)_3_.5H_2_O were added to 60 mL of an ethanol/water (1:1 v/v) mixture in the presence of 1.836 g PVP40T. After 10 minutes of stirring, 12 mL of Na_2_S_2_O_3_ solution was injected by a syringe pump with a flow rate of 0.88 mL/min. The reaction was carried out at room temperature. The suspension solution changed from yellow to lime-green color, indicating the formation of Ni(OH)_2_. Eventually, the final samples were collected after being thoroughly washed with water and ethanol for 3 times. Stable oxides were obtained after annealing these nanocages at 400 °C for 3 hours.

## Optimization of the CNBO synthetic route

The Na_2_S_2_O_3_ concentration and reaction time were optimized to optimally tune composition, morphology, and size of the synthesized materials.

Indeed, since in the CEP process the etching of the Cu_2_O core and the precipitation of Ni and Bi hydroxides take place concurrently, the concentration of Na_2_S_2_O_3_ and the reaction time are critical factors that determine the core size, the composition and the actual morphology of the core-shell particles. The first synthetic attempts were conducted with reaction times of 10 minutes and injecting 1 M Na_2_S_2_O_3_. However, the high concentration of the reducing agent (S_2_O_3_^2-^) caused a fast etching of Cu_2_O, thus resulting in the complete destruction of the sphere-like Cu_2_O structures. In turn, the abundant release of OH^-^ caused uncontrolled precipitation of Ni(OH)_2_ and Bi(OH)_3_. Because of these two phenomena, no core-shell structure was obtained with this methodology (**Figure S3a**). The outcome of the experiment indicated that milder reaction conditions (*i.e.,* lower concentration of the reducing agent and shorter reaction times) would increase the chance of success. Keeping the same Na_2_S_2_O_3_ concentration while shortening the reaction time to 3 minutes yield better results: Cu_2_O spheres are indeed still visible after reaction, although we did not obtain a clear core-shell structure (**Figure S3b**). Indeed, a large island of isolated Ni and Bi hydroxides were formed and the coverage of Cu_2_O in the mixed Ni-Bi oxide was not homogeneous. With halved Na_2_S_2_O_3_ concentration (*i.e.*, 0.5 M) and the reaction time set at 3 minutes, Ni and Bi were deposited more homogeneously on Cu_2_O spheres. However, Cu_2_O was still etched too fast, as hollow nanostructures were obtained instead of the desired core-shell structure (**Figure S3c**). A further decrease in the concentration of the reducing agent was therefore necessary to achieve a CNBO core-shell structure, as described in the following paragraph.


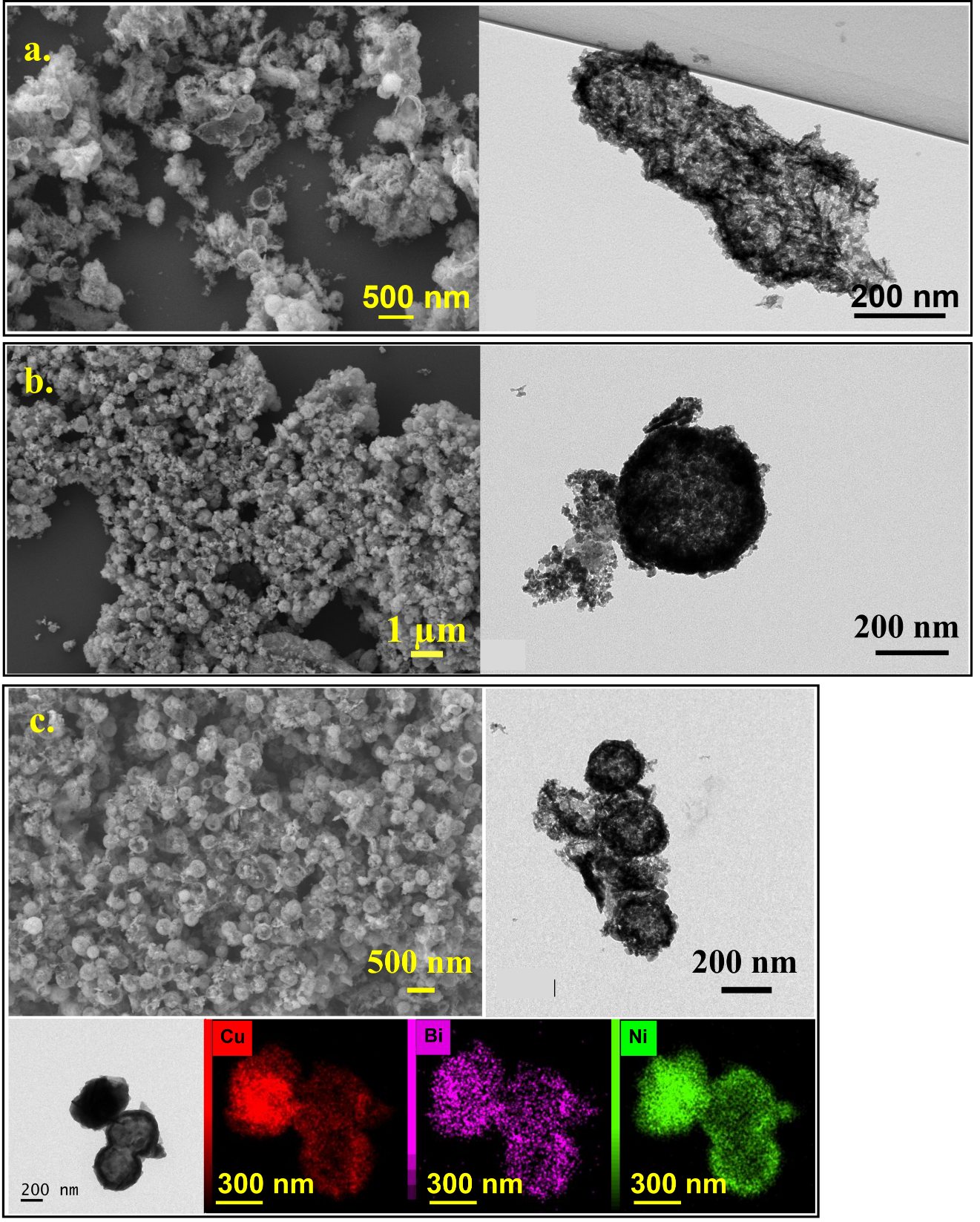


**Figure S3.** Morphologies obtained in the process of optimization of the CNBO synthesis, *i.e.,* testing different sodium thiosulfate concentration and reaction time. (**a**) 1 M Na_2_S_2_O_3_ and 10 minutes; (**b**) 1 M Na_2_S_2_O_3_ and 3 minutes and (**c**) 0.5 M Na_2_S_2_O_3_ and 3 minutes.

## Optimized CNBO synthesis and preliminary characterization

Finally, the optimal synthetic conditions were found in a Na_2_S_2_O_3_ concentration of 0.1 M and the reaction time of 3 minutes. This method yielded what seems to be a core-shell structure (**Figure S4a**) in which Ni and Bi are mainly and evenly distributed on surface (*i.e.*, the shell) while Cu constitutes the bulk of the particle (*i.e.*, the core) (**Figure S4b-g**). Interestingly, sulfur was also detected during the EDS analysis (**Figure S4e**). Its presence is tentatively assigned to the nature of the reducing agent (sodium thiosulfate), whose anions might have been trapped into the structure during the precipitation. From the cross-sectional EDS (**Figure S4h**), Ni and Bi are distributed at the edges of the particle while Cu is mainly located at the center. This supports the core-shell nature of the sample, with a shell composed of nickel and bismuth oxide, and a CuO_x_-based core. Similar results were obtained also for the control sample (CNO, **Figure S5**). However, the line EDS analysis showed significant noise in the distribution of Ni and Bi, making it inconclusive in determining the core and shell composition and structure. As discussed in the main text, a higher resolution in imaging and EDS mapping revealed a complex structure that can be more accurately defined as a nanocage than a shell (**Figure 1f**).


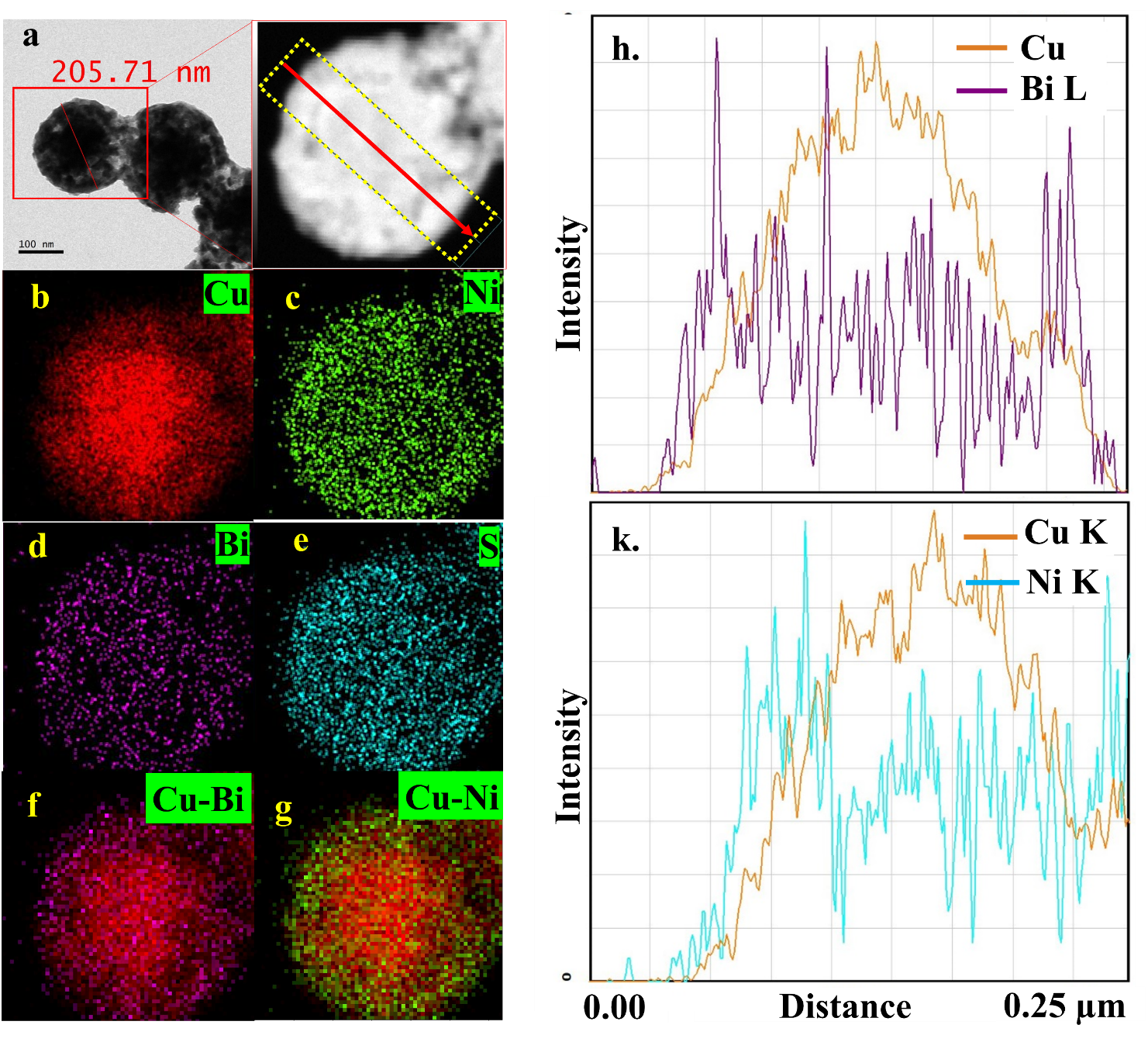


**Figure S4.** Morphological characterization of the optimized CNBO synthesis, which employs 0.1M Na_2_S_2_O_3_ and 3 min as the reaction time. **(a)** TEM image and **(b-g)** related EDS mapping, **(h-k)** EDS line profiles of Cu, Ni and Bi.


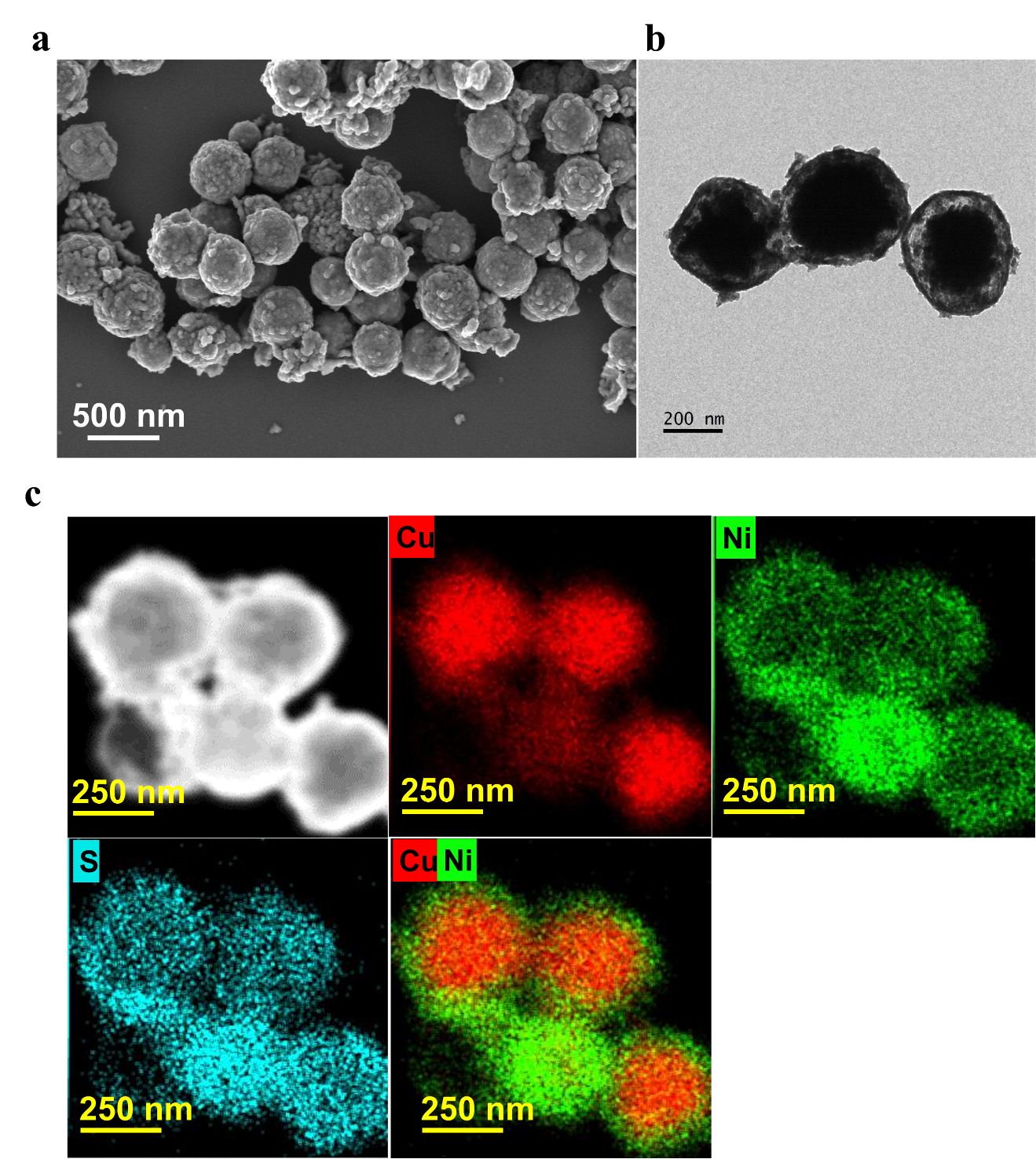


**Figure S5.** Morphological characterization of the control sample CNO, obtained according to the optimized route described for CNBO but without the addition of the Bi precursor. **(a,b)** SEM and BF-TEM images of the sample. (**c**) STEM-ADF and related STEM-EDS maps.

The peak areas obtained in the STEM-EDS spectrum (**Figure S6**) were used to calculate the atomic fraction within the chosen particles in **Figure 1f**. It is noteworthy that the atomic ratio between Cu:Ni:Bi was similar to the ratio obtained by ICP, indicating Ni and Bi are just deposited on Cu template and did not form separate phases (**Table S1**).


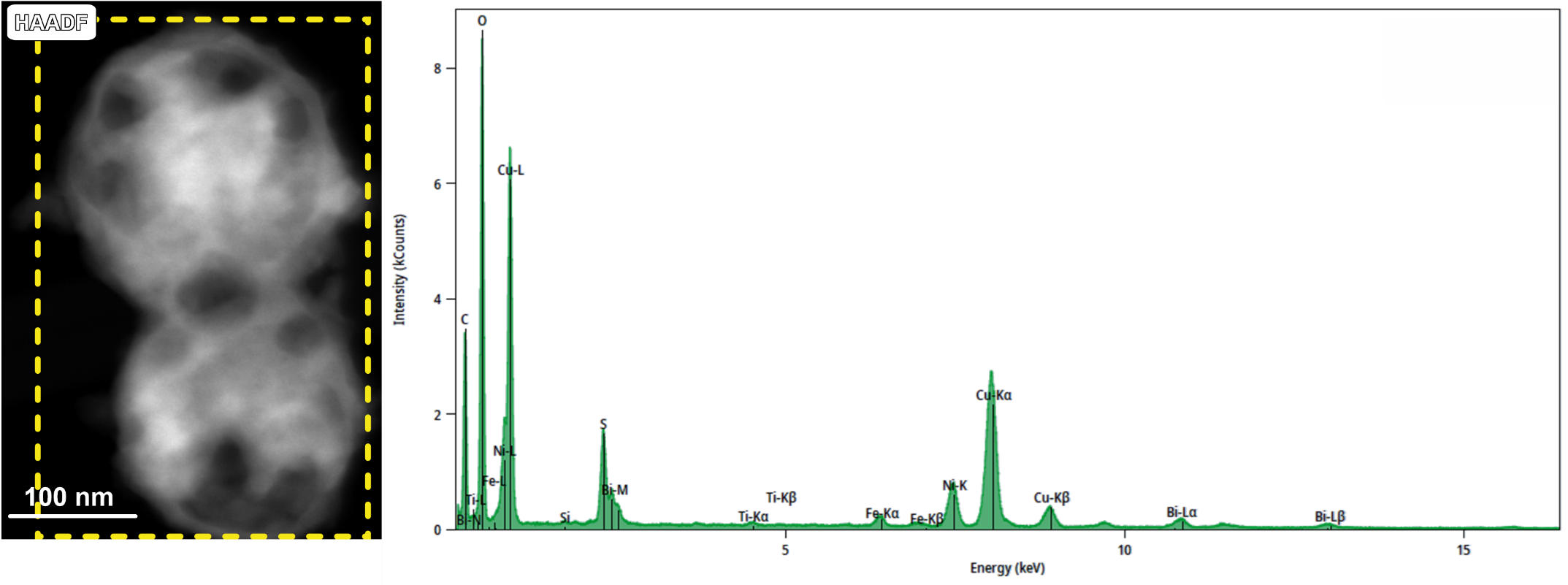


**Figure S6.** EDS spectrum of CNBO obtained by STEM-EDS

**Table S1.** Atomic ratio of metallic ions in CNBO, including nominal values from the synthesis and results from inductively coupled plasma optical emission spectroscopy and STEM-EDS quantification.

| **Elements** | **Synthesis (by design)** | **ICP-OES** | **STEM-EDS** |
| --- | --- | --- | --- |
| Cu | 91.6 | 78.5 | 76.72 ± 2.66 |
| Ni | 6.7 | 17.8 | 19.15 ± 2.21 |
| Bi | 1.8 | 3.7 | 4.13 ± 0.64 |

## Surface composition of samples (XPS analyses)

**Figure S7** shows the Cu, Ni, and Bi high-resolution XPS spectra collected on the three samples (from top to bottom: CuO, CNO, and CNBO). In all materials, Cu exists mainly as Cu^2+^, as expected considering the thermal treatment all samples have been subjected to. In CNBO, Bi 4f peaks centered at a binding energy of 159.3 and 164.6 eV (±0.2 eV) correspond to Bi(III) in Bi_2_O_3_. The area under the peaks can be used to estimate the relative concentration of elements. XPS indicates a Cu:Ni atomic ratio of approx. 40:32 (*i.e.,* 1.25:1) in CNO while the ratio between Cu:(Ni+Bi) is 41.5:31.9 (*i.e.,* 1.3:1) in CNBO. Noteworthy, the ratio of (Ni+Bi)/Cu is higher than what obtained by ICP-OES and STEM-EDS measurements, thus implying that Ni and Bi are mainly located on the surface of catalyst, as expected considering the synthetic mechanism of CEP.


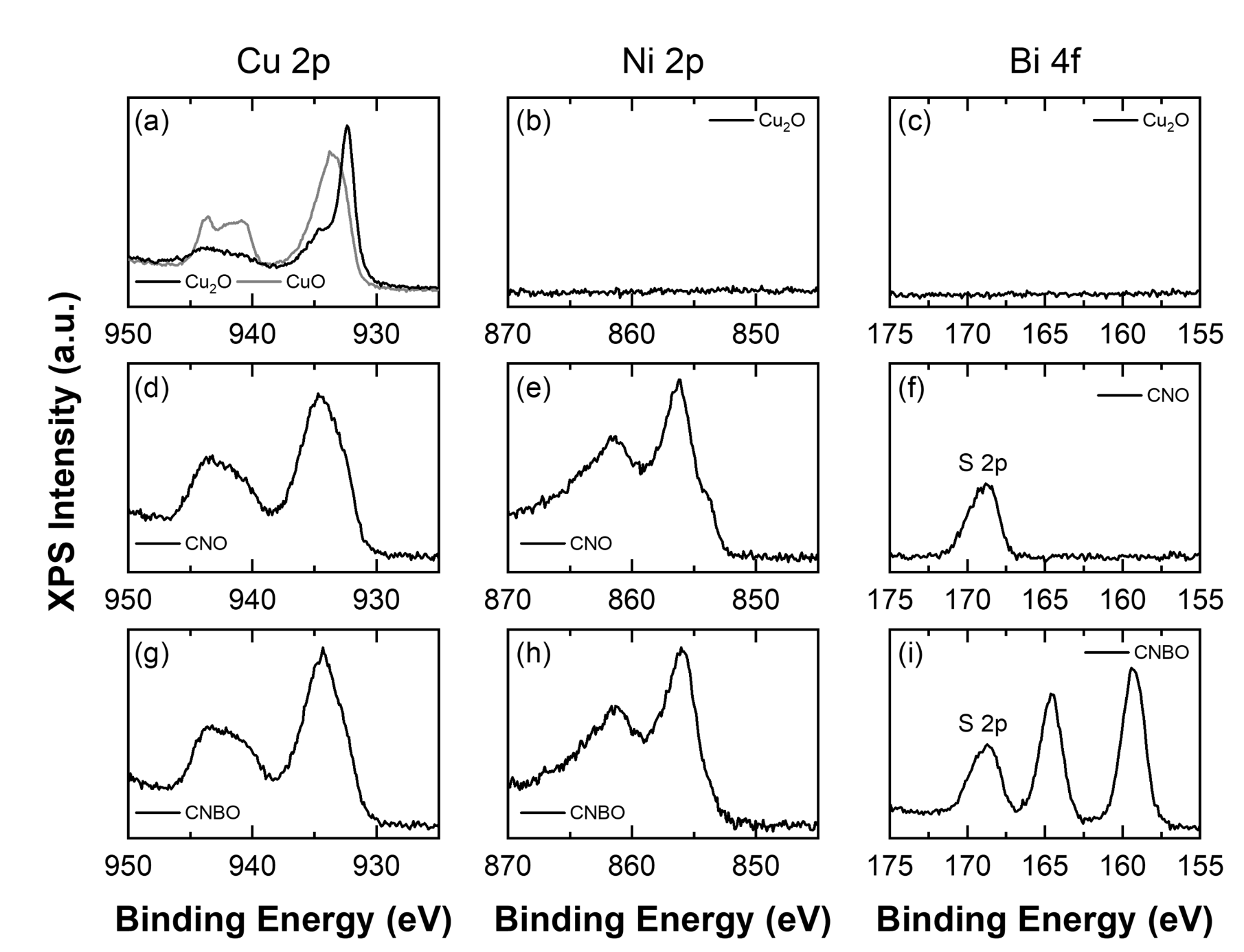


**Figure S7.** XPS spectra of (**a, b, c**) CuO and Cu_2_O; (**d, e, f**) CNO, and (**g, h, i**) CNBO.

# Electrochemical testing in H-cell – Additional details

A basic electrochemical investigation of the samples was conducted in a three-electrode configuration in the H-cell depicted in **Figure S8**. An aqueous 1 M KOH solution, with or without 0.1 M glycerol, was used as electrolyte. The prepared electrodes, a standard Hg/HgO, and a Pt coil were used as the working, reference, and counter electrodes, respectively. An Anion Exchange Membrane (AEM, Fumasep FAB-PK-130, from FuelCellStore), activated according to the producer guidelines, was used to separate the anodic and cathodic compartments. All the tests and electrochemical experiments were conducted at room temperature.


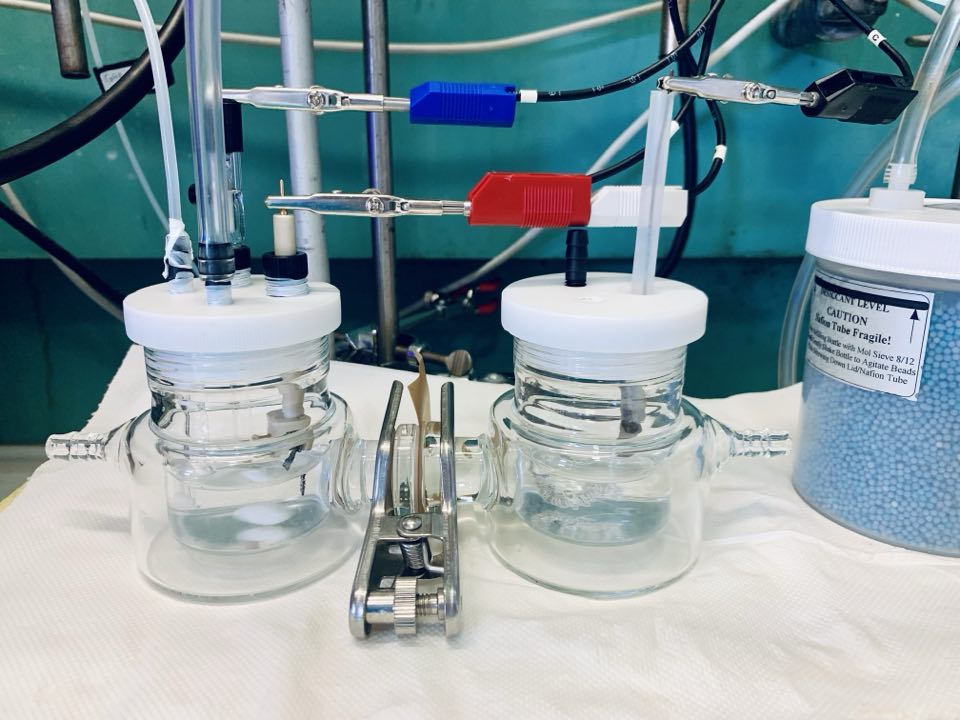


**Figure S8.** Electrochemical setup used for GEOR and OER testing under a three-electrode, H-cell configuration.

At first, the working electrodes were activated by multiple CV cycles upon the achievement of overlapped traces. As presented in **Figure S9**, the current delivered by the carbon substrate (tested as operative blank, fabricated upon deposition of a mock catalytic ink containing Nafion but no active material) is negligible and does not contribute to the overall performance of the catalyst. As predicted, CuO delivers low current under both OER and GEOR conditions. No particular feature can be spotted in CuO electrochemical traces. Conversely, the presence of nickel in CNO and CNBO results in the typical Ni^2+^/Ni^3+^ redox peaks (at 1.48 and 1.32 V vs RHE, respectively)^[6]^. A few considerations can be made on these peaks. First, these features are visible only under OER conditions (*i.e.*, in the absence of glycerol). Considering the earlier onset of GEOR (compare full and dashed curves in **Figure S9**, for example), this might indicate that glycerol reacts prior to the Ni^2+^ oxidation to Ni^3+^ (*i.e.*, before NiOOH formation, the species renowned for being the active site of Ni-based catalysts in OER). However, further *in-situ* Raman experiments, detailed in the dedicated sections, support an indirect oxidation mechanism for glycerol, with Ni^3+^ (in the form of NiOOH) acting as a mediator. Second consideration: compared to CNBO, CNO shows a larger Ni^2+^/Ni^3+^ peak area, indicating the oxidation of a higher number of Ni^2+^ species. This is consistent with the XPS results, exhibiting a mixed Ni^2+^/Ni^3+^ distribution on the surface of CNO while highlighting a large majority of Ni^3+^ species in the case of CNBO.

Probably because of glycerol diffusional limitations at high reaction rates (*i.e.*, high current densities), the current delivered by CNBO exhibits a peak at high potential values. It is also interesting to notice that the reverse scan (cathodic direction) of the CV collected onto CNBO presents a hysteresis, with the anodic and cathodic branches crossing around 1.47 V vs RHE. We tentatively assign this behavior to the possible oxidation of adsorbed intermediates, formed upon the anodic scan, and still present on the surface after the reversing of the potential scan.


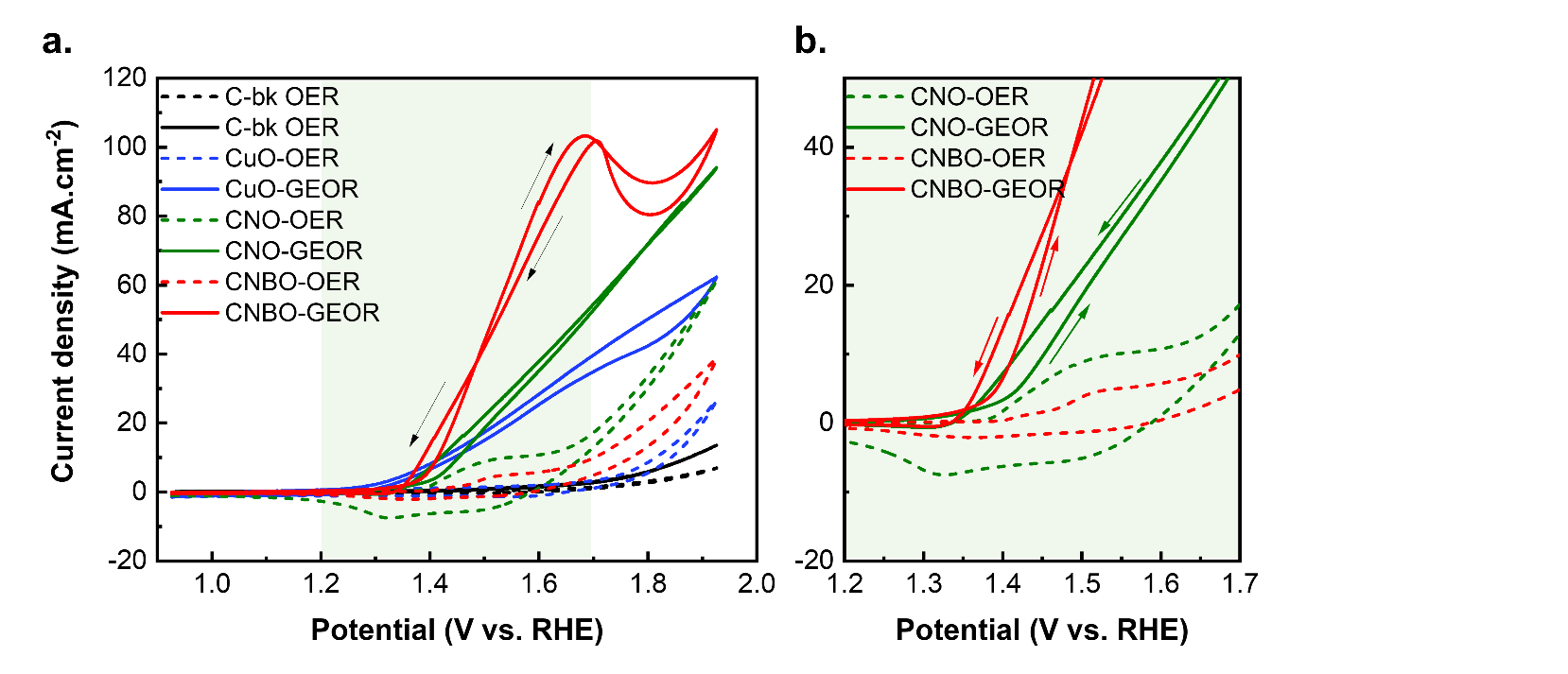


**Figure S9. (a)** 10^th^ CV scan collected on bare carbon paper (blank), control samples (CuO and CNO), and the actual catalyst (CNBO) under OER and GEOR conditions; **(b)** magnification of the CV scans of CNO and CNBO under OER and GEOR conditions in the potential window from 1.2 V to 1.7 V. CV parameters: scan rate = 100 mV s^-1^; E_Step_: 1 mV.

GEIS was performed to analyze the impedance of the system. The circuit depicted in the inset of **Figure S10a** was used to fit the data, which followed a two-semicircle profile in the GEIS. Accordingly, R_1_ is assigned to the system resistance (R_u_), representing a combination of the electrolyte resistance, the contact resistance between the catalysts and the current collector, and the resistance of the electrode matrix. The R_1_ values collected for CNBO, CNO, and CuO are listed in **Figure S10b.** These values are close to 0, so IR correction can be ignored in this three-electrode system.


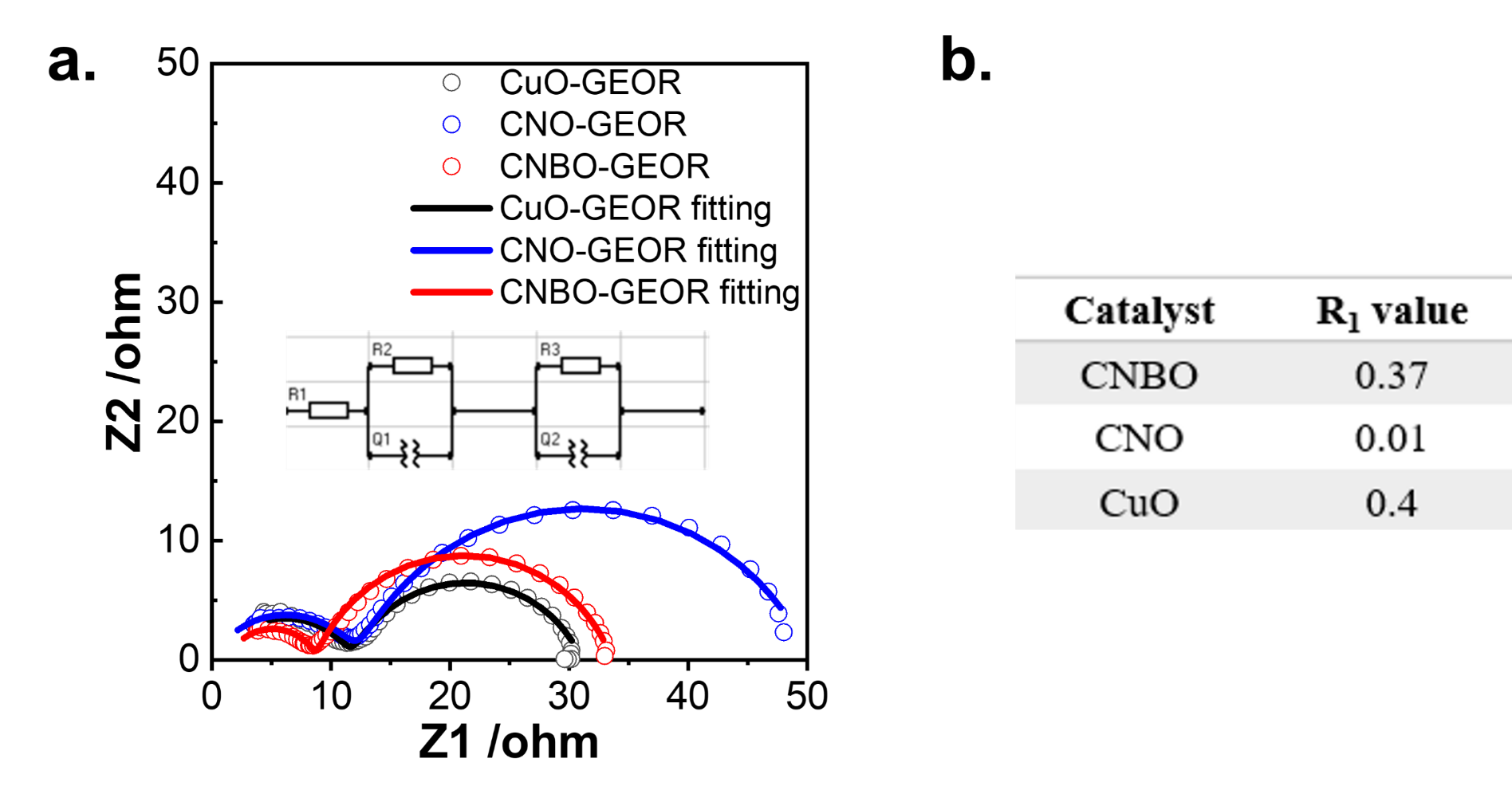


## Figure S10. Nyquist plots (a) and fitted R_1_ data (b) of CuO, CNO and CNBO under GEOR condition at the current of 2.5 mA.

# GEOR and CO_2_RR products analysis

## GEOR products analysis

Glycerol and its liquid/soluble oxidation products (*i.e.*, glyceric acid, dihydroxyacetone, glycolic acid, formic acid, …) have been separated by means of High Performance Liquid Chromatography (HPLC) and inline detected and quantified by both a Diode Array Detector (DAD, operating at λ = 210 nm) and a Refractive Index Detection (RID, operating at 35°C). An Agilent Hi-Plex-H 300 x 7.7 mm column, heated at 60°C, was used to separate products in post-reaction electrolytes (injected sample volume equal to 40 μL). The HPLC mobile phase was an aqueous H_2_SO_4_ (5 mM) solution, pumped at a flow rate of 0.6 ml/min.

Prior to analyzing the post-reaction samples, multiple injections of standard solutions at known concentration of glycerol and its common oxidization products were used to generate calibration lines.


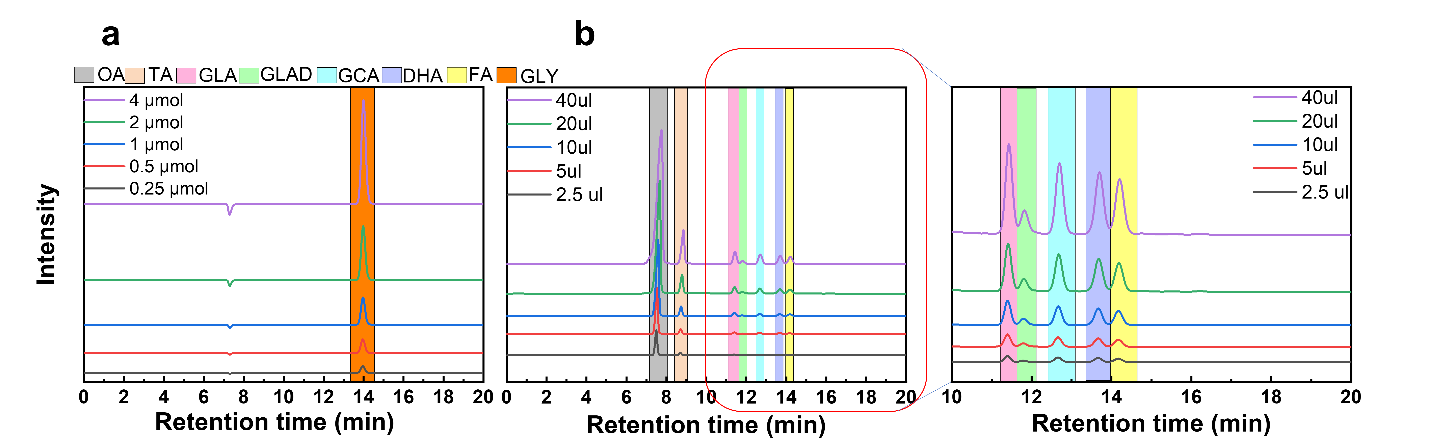


**Figure S11.** Sample HPLC spectra, obtained upon the injection of standard glycerol and GEOR products during the calibration procedure. (**a**) Typical glycerol (GLY) HPLC trace (RID); (**b**) HPLC trace obtained upon the injection of a mixture of glycerol and the most common GEOR products (glyceraldehyde (GLAD), dihydroxyacetone (DHA), glyceric acid (GLA), glycolic acid (GCA), formic acid (FA), tartronic acid (TA), oxalic acid (OA))(DAD, DAD-λ = 210 nm).


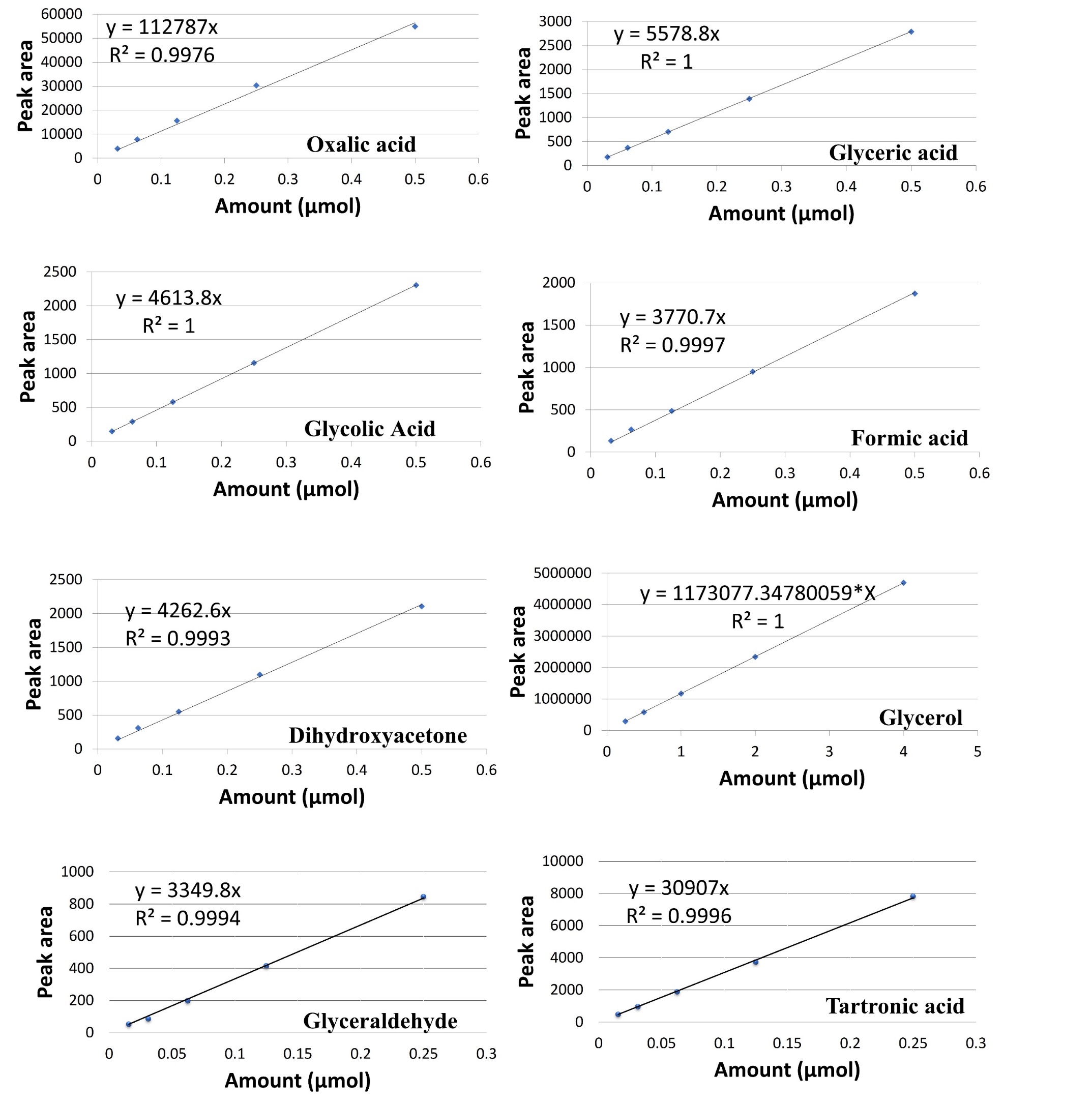


**Figure S12.** Glycerol calibration line plotted using data from the RID detector and GEOR products calibration lines plotted based on data obtained from the DAD detector.

The Faraday efficiency (FE, calculated as %) of the product formation can be determined according to a formula derived from Faraday equations:

*FE(%)=*$\frac{Q_{products}}{Q_{total}}=\frac{\sum z\times n\times F}{Q_{total}} \times100\%$

where Q_products_ is the amount of charge stocked into the products under study, Q_total_ is the total charge passed through the electrode, *z* is the number of electrons required per product, n is the moles of the product formed, F is the Faraday constant (96485 C mol^-1^).

The ratio between the moles of carbon stocked in GEOR products and the converted amount of carbon (based on consumed glycerol), defined as carbon balance, can be calculated using the following equation.

*Carbon balance (%) =* $\frac{{C3}_{prod}\times3 + {C2}_{prod}\times2+{C1}_{prod} x1}{\left( {Gly}_{i} - {Gly}_{f} \right)\times3}\times100\%$

where Gly_i_ and Gly_f_ represent the initial and final glycerol moles, respectively, while C1_prod._, C2_prod._, C3_prod_ are the moles of one-, two-, and three-carbon products generated during GEOR.

## CO_2_RR products analysis

Liquid/soluble CO_2_RR products (*e.g.,* formic acid) have been detected and quantified by HPLC with the same method reported for GEOR.

Gaseous CO_2_RR products (*i.e.*, parasitic H_2_, CO, CH_4_, C_2_H_4_...) and GEOR parasitic by-product (O_2_) have been identified and quantified using a SRI 8610C gas chromatograph (in Multiple Gas Analyzer configuration #5) equipped with a thermal conductivity detector (TCD) and flame ionization detector (FID) coupled with a methanizer. Prior to experiments, detectors have been calibrated using an *ad-hoc* cylinder mixture.


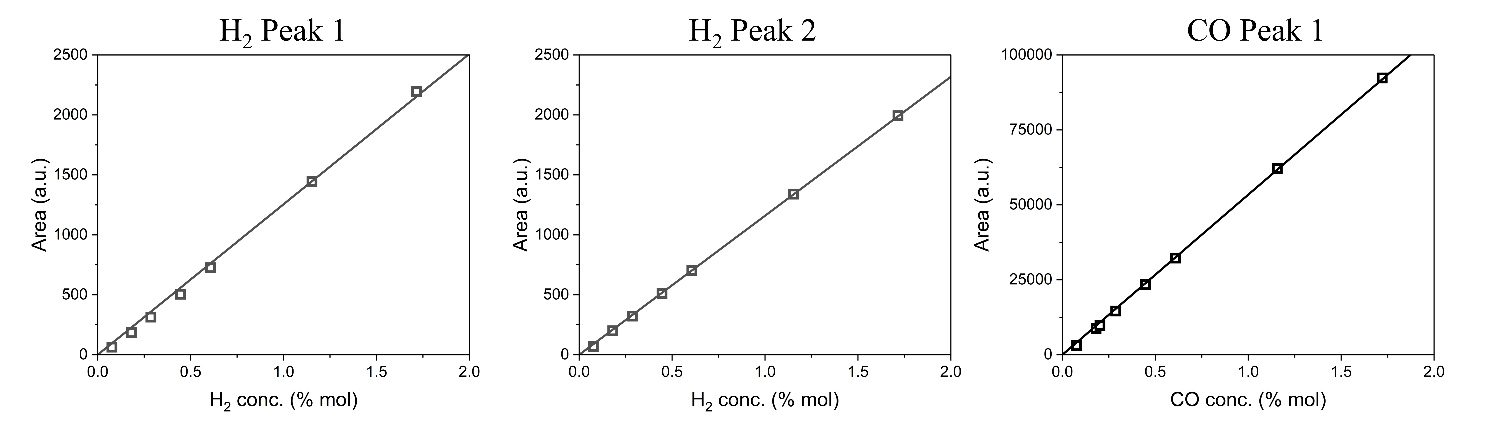


**Figure S13.** Gas products (H_2_ and CO) calibration lines. The relative peak area is plotted vs. that of the calibration gas standards.

## Checking OER activity over CNBO - Gas chromatography

The above detailed GC setup was connected to the H-cell with argon gas (used in this case as a carrier from the cell to the GC itself) continuously purged into the anolyte at a flow rate of 5 ml/min. The H-cell outlet gas was sampled and analyzed at 0 minutes (blank run) and after 60 minutes of GEOR over CNBO at 50 mA cm^-2^. The oxygen peak analyzed at 60 minutes shows no obvious difference from the background, suggesting no oxygen was formed under reaction conditions.


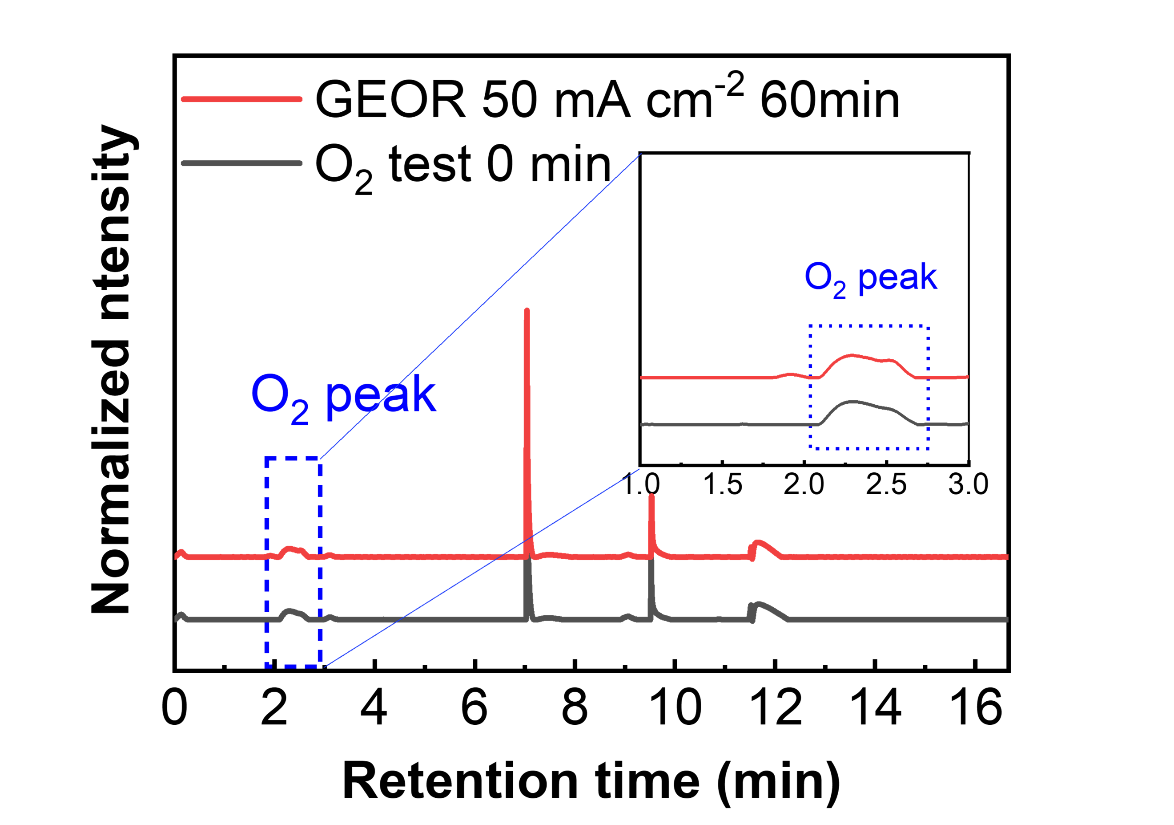


**Figure S14.** GC chromatograph obtained at different times (0 and 60 minutes) of a chronopotentiometric scan (50 mA cm^-2^) on CNBO under GEOR conditions.

# Reaction pathway

A tentative reaction pathway for GEOR on CNBO can be proposed based on the results of specific experiments in which the observed GEOR products have been individually oxidized under alkaline conditions. In particular, the individual oxidation of glyceric, glycolic and oxalic acid have been carried out and the reaction products analyzed. The HPLC chromatographs (**Figure S15a-c**), collected on the post-reaction electrolytes of the different reactions, show that glyceric acid undergoes oxidation to form glycolic acid and formic acid. The alcohol group of glycolic acid is further oxidized to a carboxylic group, resulting in the formation of oxalic acid, with formic acid as a product when C-C cleavage happen. No products are instead detected upon oxalic acid oxidation. Considering that GEOR on CNBO yields low FE in glyceric acid and glycolic acid and high FE in formic acid and considering the results obtained when individually oxidizing intermediates, we can tentatively infer the reaction mechanism reported in **Figure S15d**, *i.e.*, glycerol → glyceric acid → glycolic acid → formic acid.


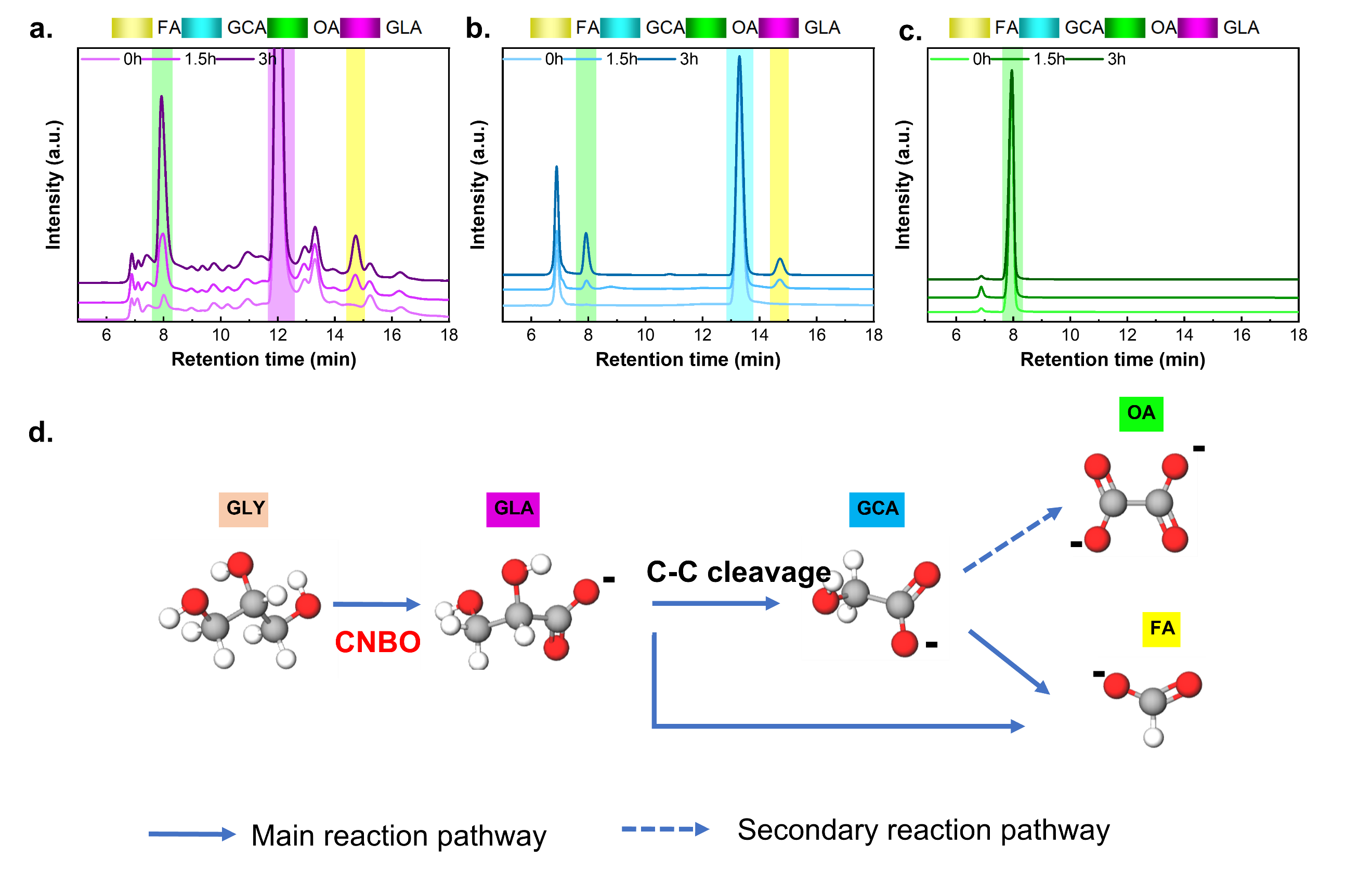


**Figure S15.** HPLC chromatograms obtained upon the oxidation of different GEOR intermediates, namely **(a)** glyceric acid, **(b)** glycolic acid, **(c)** oxalic acid, at 10 mA cm^-2^, **(d)** reaction pathway of GEOR on CNBO.

# Electrochemical performance of control materials

The one hour-long chronopotentiometric traces recorded on CuO and CNO are reported in **Figure S16a-b**. The potentials for GEOR to achieve the current densities of 10, 25, and 50 mAcm^-2^ over CuO, CNO, and CNBO are summarized in **Figure S16c.** The potential required to reach the current density of 50 mA cm^-2^ over CBNO and CNO is 340 and 250 mV lower than that of CuO, respectively. This is consistent with the composition of the catalysts and the expected increase in activity when Ni and Bi are inserted into the catalytic matrix. At applied current densities of 25 and 50 mA cm^-2^, the required potentials for GEOR over CuO were significantly higher than that of CNO. These findings suggest that despite CuO might be an acceptable GEOR catalyst under mild conditions (10 mA cm^-2^), its performance deteriorates at high current densities, likely due to its instability, stemming from the easy oxidization of the oxide to soluble CuO_2_^2-^ in strong alkaline environments^[7]^.

The GEOR product distribution yielded by CuO and CNO is depicted in **Figure S16d-e**. Similar to what observed for CNBO, the carbon balance closes around 100% and formic acid contributed as the main product with high FE (generally, > 70%). It is interesting to notice that the total FE does not achieve unity for these CuO and CNO materials, especially in the case of CuO. Considering that our analytics detect all C-based products stemming from GEOR and that the carbon balance consistently closes at 100%, possible reasons for this behavior may be the formation (and consequent dissolution) of CuO to CuO_2_^2-^ or carbon substrate oxidation. GEOR catalyzed by CNO produces FA with slightly higher FE (≈85%) compared to CNBO (≈80%). This consistence with the impact of Bi on protecting C-C bond from cleavage, reducing the forming of 1C product^[8]^.


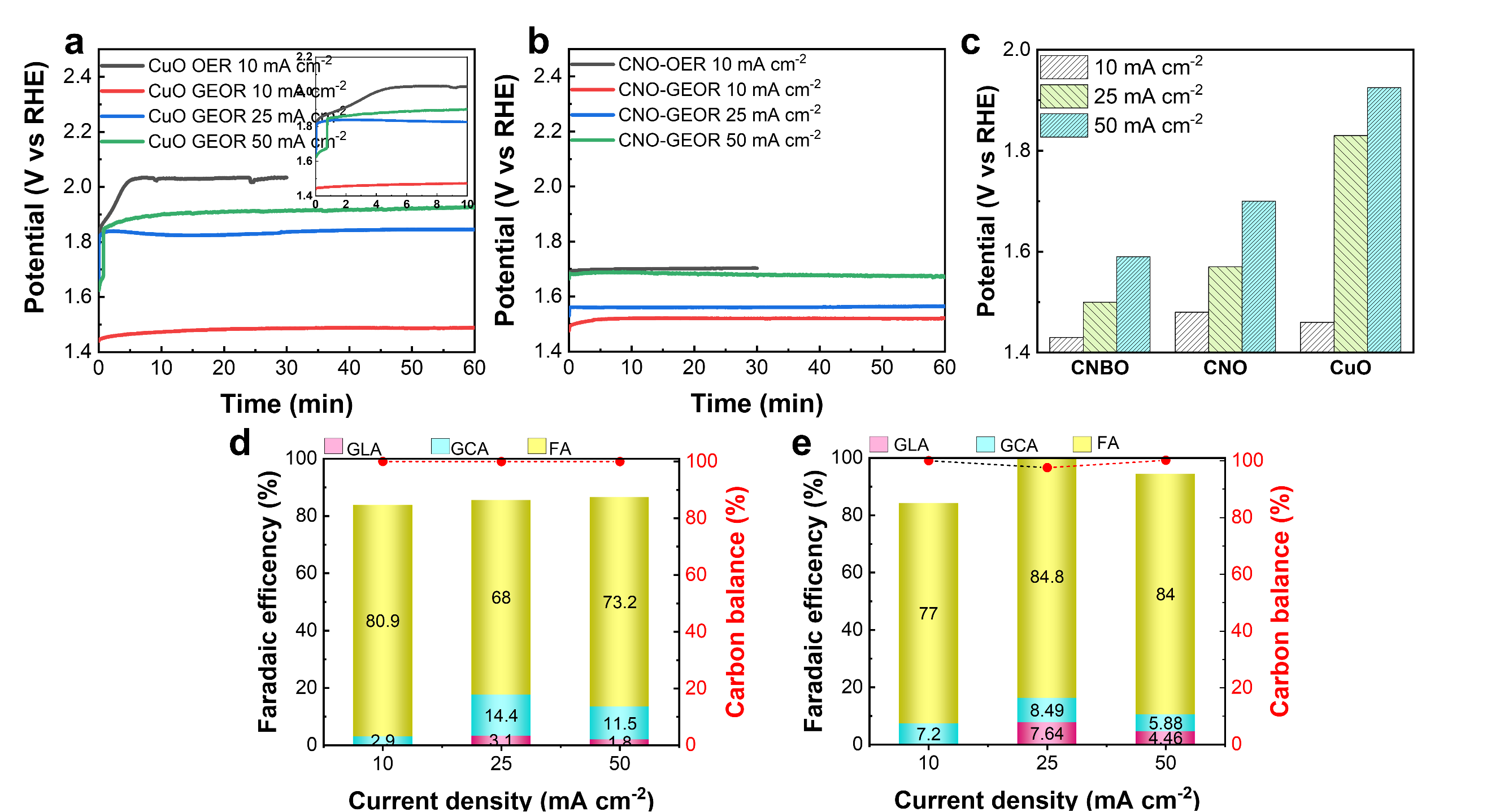


**Figure S16.** Chronopotentiometric traces collected on **(a)** CuO and **(b)** CNO at different applied current densities under both OER and GEOR conditions; **(c)** anodic potentials required for GEOR at different reaction rates (*i.e.*, current densities, namely 10, 25 and 50 mA cm^-2^) on CuO, CNO and CNBO; GEOR product distribution obtained on **(d)** CuO and **(e)** CNO.

An additional control experiment was carried out to evaluate the role of the CuO core in CNBO. Hollow CNBO particles were synthesized following the same method used for CNBO core-shell-like particles but completely leaching the Cu-based core by a 30 minutes-long immersion in 1 M Na_2_S_2_O_3_. STEM-EDS was conducted to confirm the hollow sphere structure of the material (**Figure S17**).

**.
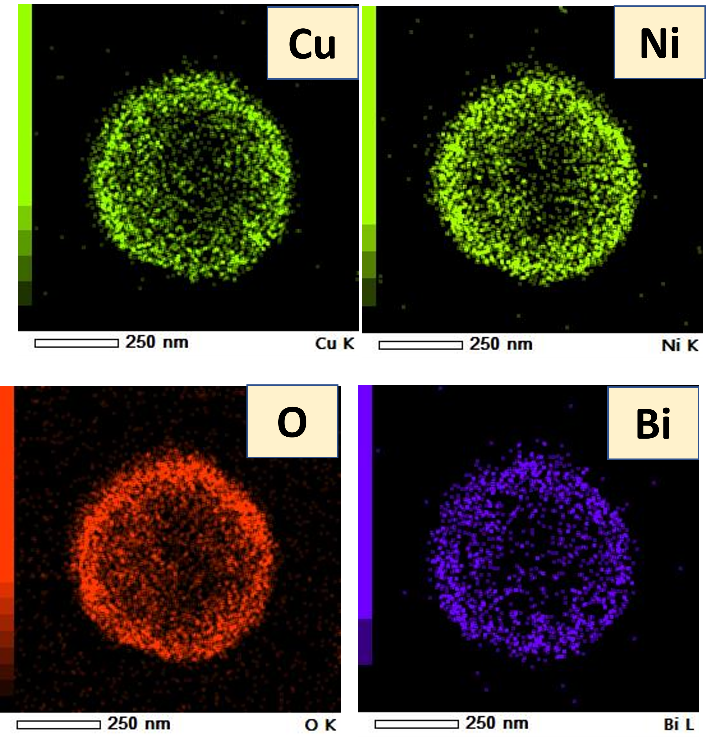
**

**Figure S17.** STEM-EDS maps of CNBO hollow sphere.

With the same mass loading of 0.5 mg cm^-2^, CNBO exhibited better catalytic activity compared to the hollow CNBO particles, as demonstrated by the polarization curves and chronopotentiometric tests (**Figure S18a** and **b**). For example, a 40 mV increase in the potential recorded at 50 mA cm^-2^ is evident from the chronopotentiometric scan. GEIS data demonstrate that the hollow CNBO particles show a larger charge transfer resistance under GEOR conditions (**Figure S18c**), suggesting that the Cu-based core plays a pivotal role in facilitating the reaction, most likely in imparting facilitating electron transfer and increasing the overall conductivity of the catalytic structure. Finally, it is noteworthy that, despite the lower energy efficiency displayed by the hollow CNBO particles, their product distribution resembles the one observed for CNBO (**Figure S18d**). Thus, the product distribution is not altered by the absence of the Cu-based core.

**
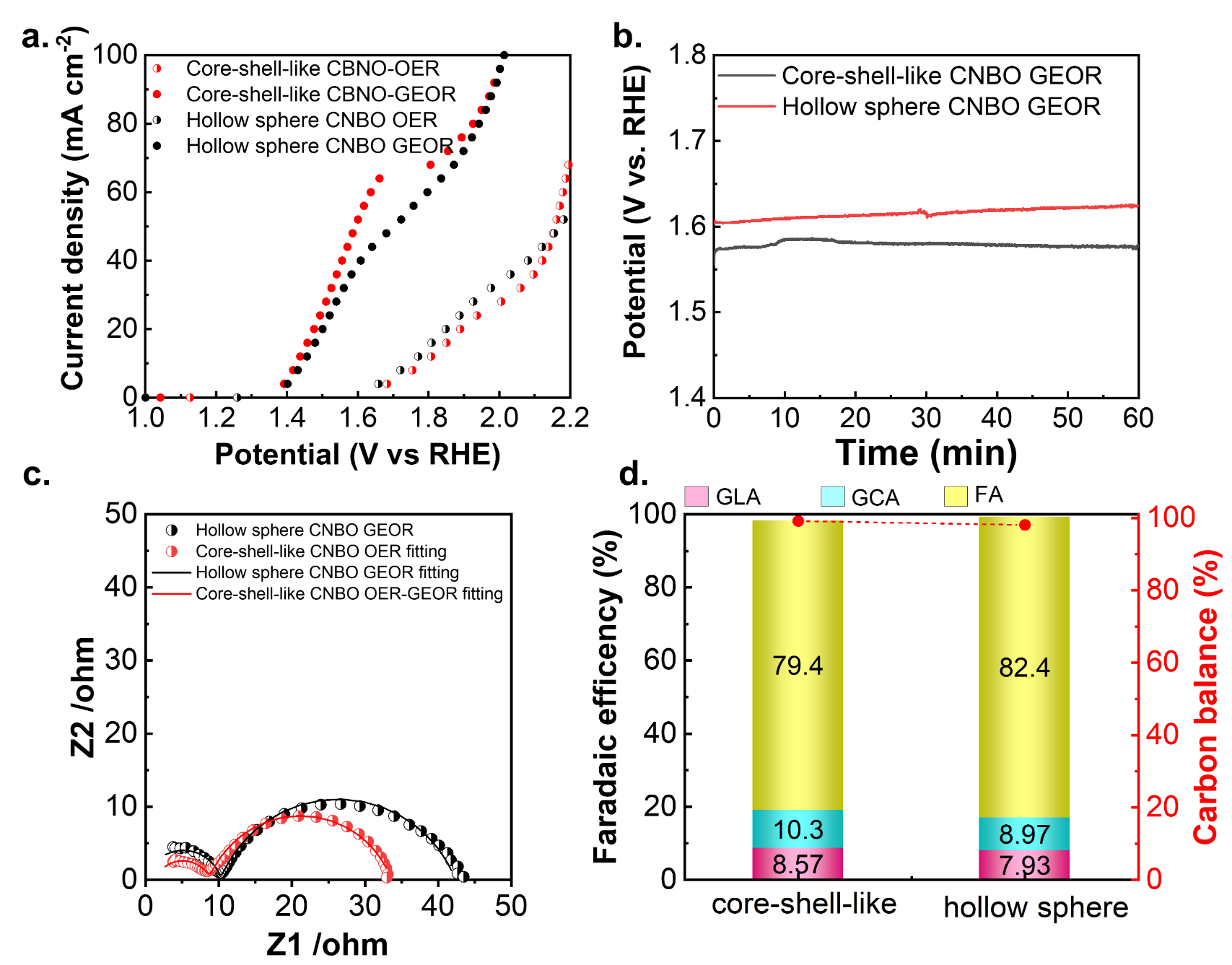
**

**Figure S18. (a)** Galvanostatic Linear Sweep Voltammetric traces collected on CNBO core-shell and CNBO hollow sphere; **(b)** Chronopotentiometric scans, **(c)** galvanostatic electrochemical impedance spectroscopy and **(d)** related product distribution obtained over CNBO core-shell and CNBO hollow sphere at current densities of 50 mA cm^-2^. GLA: glyceric acid, GCA: glycolic acid, FA: formic acid.

# Post-electrolysis analyses – Catalysts stability

Differently from the GLSV curves recorded on CNBO post-electrolysis electrodes, used CNO and CuO electrodes exhibit reduced performance. This decay suggests that CuO and CNO underwent degradation, which compromised their electrochemical performance over time. CNBO showed more resilience during the reaction and maintained its structure in comparison to CuO and CNO.


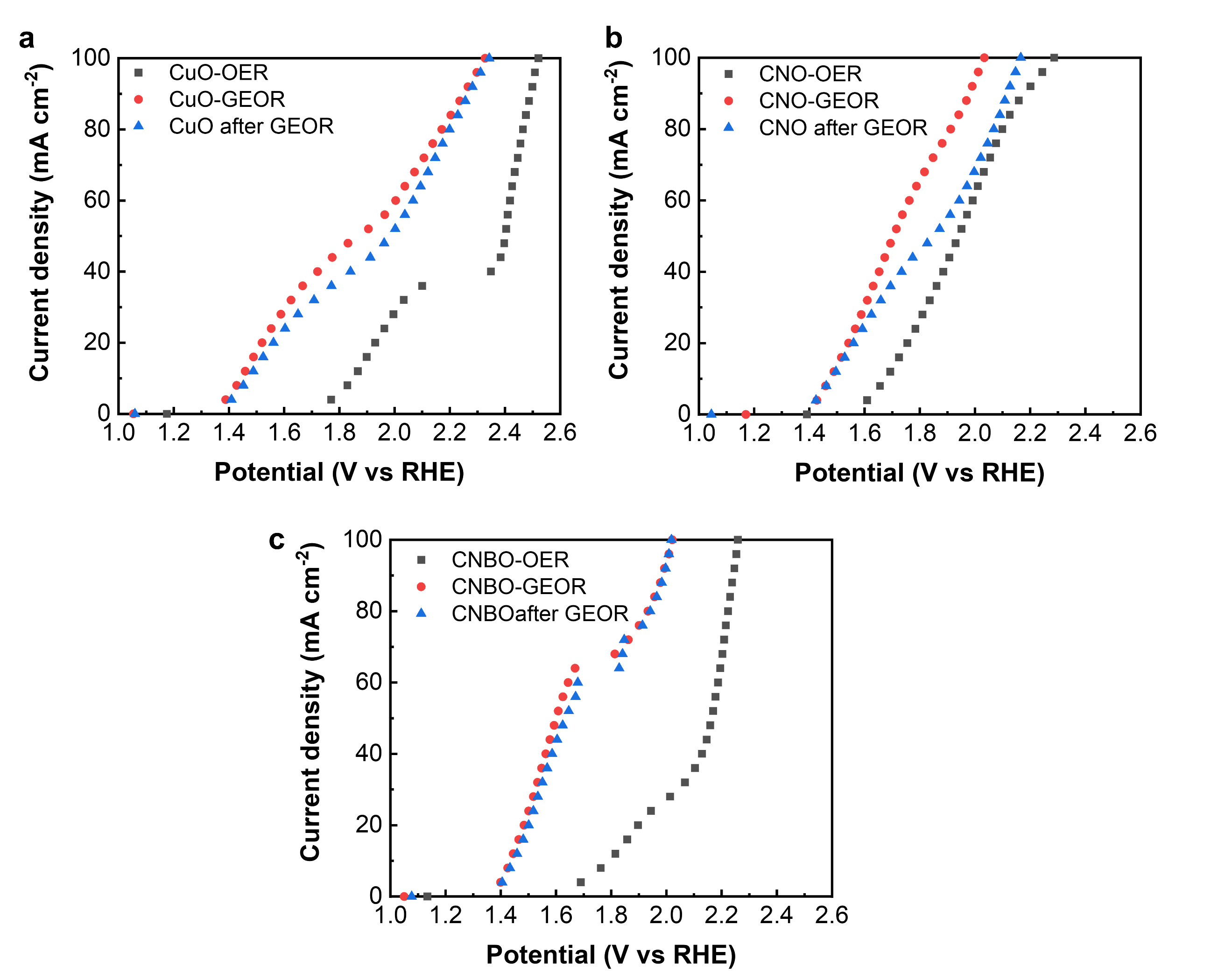


**Figure S19.** GLSV traces recorded over used electrodes (*i.e.*, one-hour long electrolysis, GEOR conditions) (**a**) CuO, (**b**) CNO, and (**c**) CNBO electrodes.

The structural integrity of the electrodes was then evaluated by observing them by SEM (**Figure S20**). While the distribution of CNO and CNBO in only slightly altered after electrolysis, the used CuO electrode exhibits many empty areas, suggesting that CuO dissolved (leaching) or underwent significant material loss (*e.g.*, by detachment) during the reaction, confirming that CuO electrodes are not stable under operating conditions.


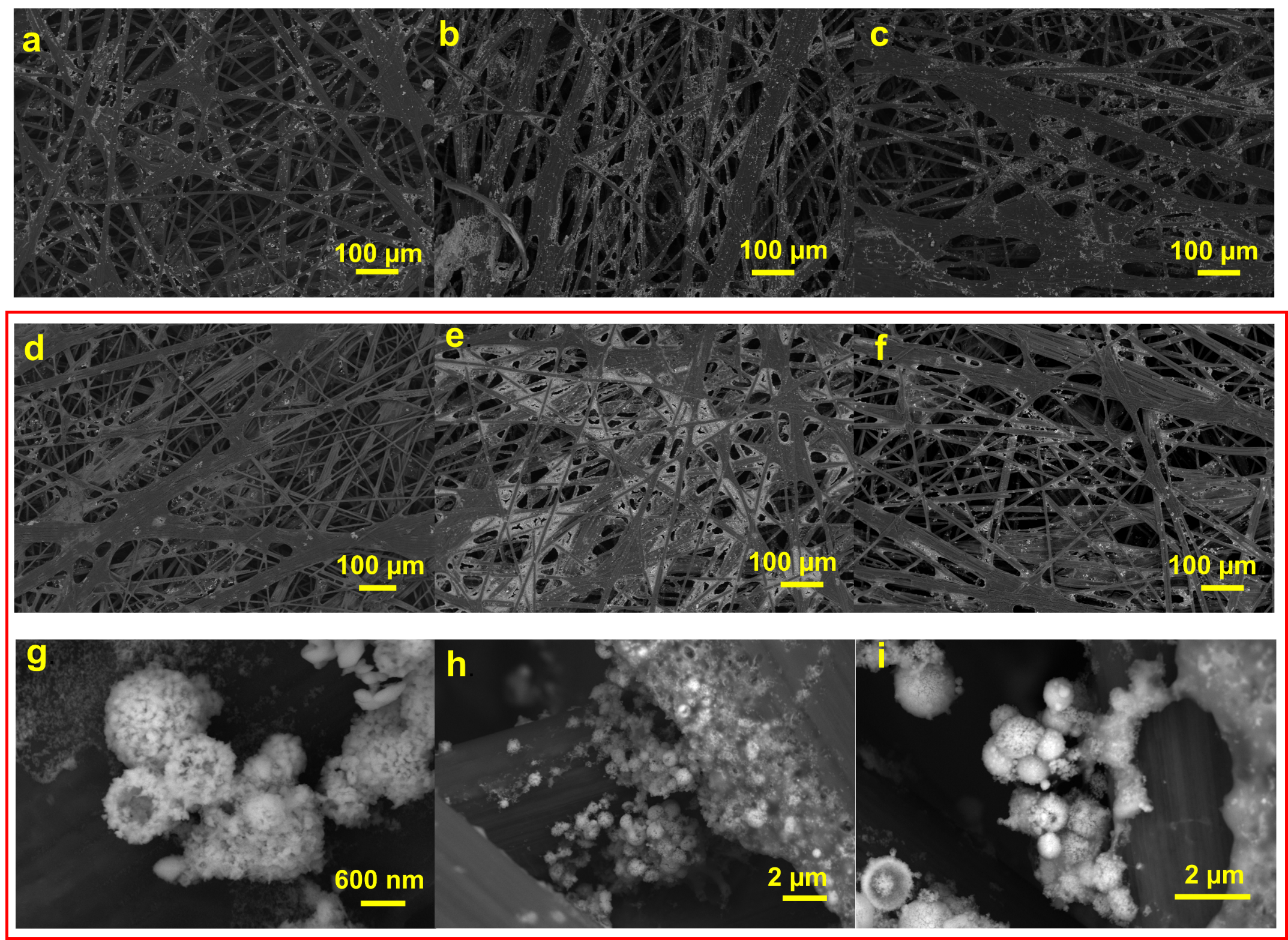


**Figure S20.** From left to right, SEM images of CuO, CNO and CNBO electrodes. **(a, b, c)** Pristine samples; **(d, e, f)** samples after electrolysis and **(g, h, i)** related high magnification views.

Chemical features of the used materials were also analyzed by *ex-situ* Raman spectroscopy, (**Figure S21**). As for the pristine samples, the most prominent peaks in three electrodes at 290, 333, and 621 cm^-1^ are assigned to CuO^[4a, 4c]^. The Raman spectra of CNO and CNBO also exhibit broad bands at 500, 780, and 1000 cm^-1^ assigned for NiO^[9]^. Due to the trace amount of bismuth in the sample, it is not easily detectable in Raman spectroscopy. Therefore, to confirm the presence of Bi, a more sensitive analytical method is required.


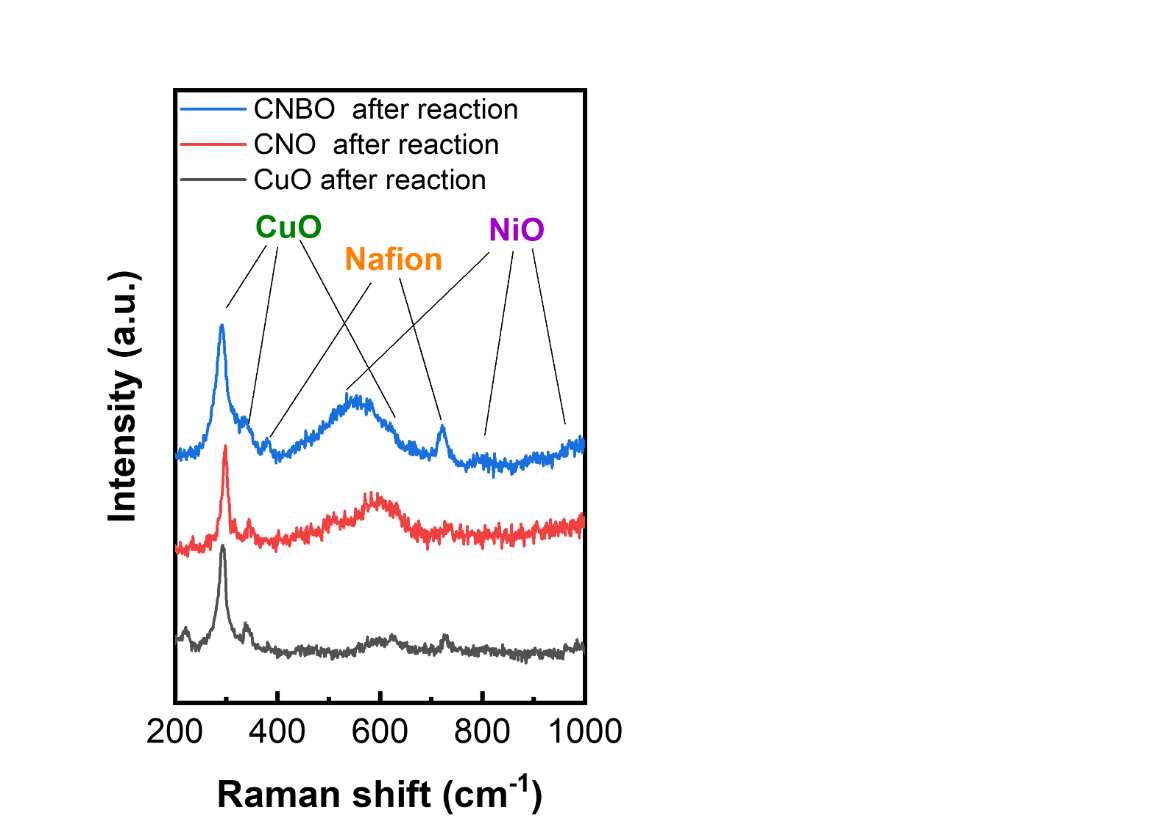


**Figure S21.** Raman spectra of CuO, CNO, and CNBO electrodes after one-hour GEOR electrolysis.

After one-hour electrolysis, the used CNBO electrode was sonicated in IPA for 10 minutes as to detach CNBO particles from the carbon paper substrate. These particles have then been collected from the solution and deposited on a TEM grid for the analysis. As depicted in **Figure S22**, CNBO particles retained their spherical morphology and Cu, Ni, and Bi were detected, thus proving the morphological and compositional stability of the material.


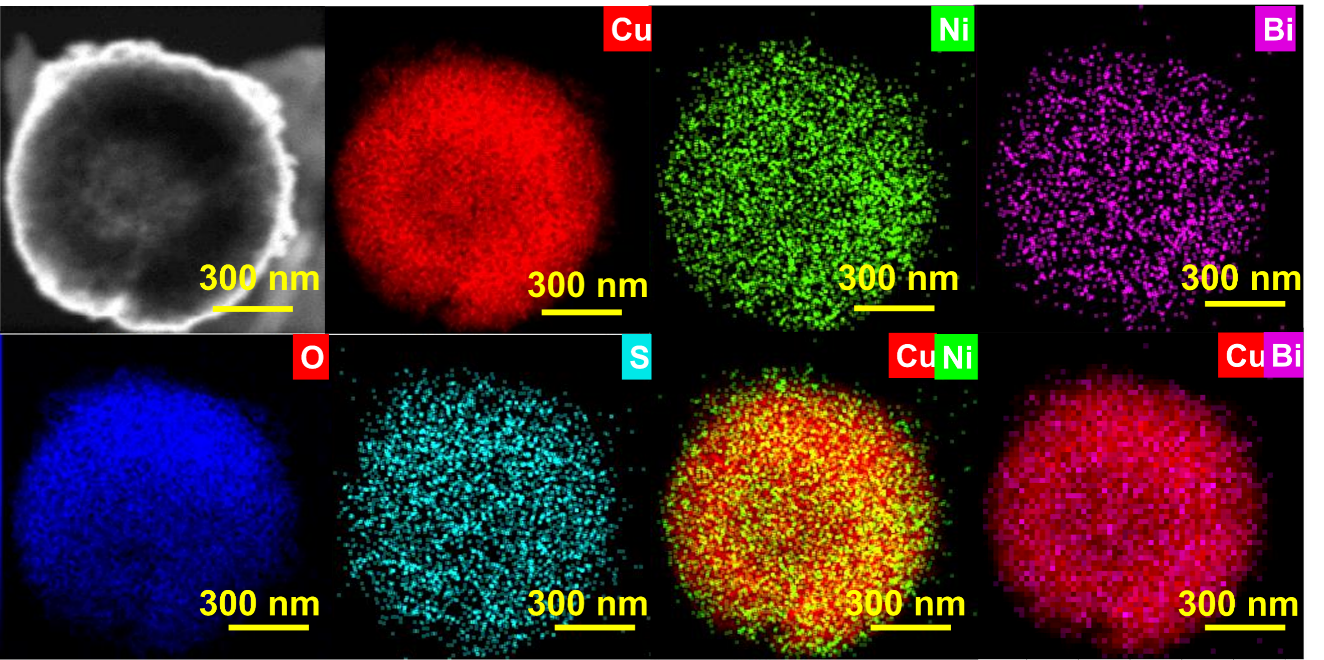


**Figure S22.** STEM-EDS image of a used CNBO (one-hour long electrolysis) with related elemental maps. Note that the apparent change in contrast and composition at the center of the particle is likely to be due to thickness effects affecting the analysis.


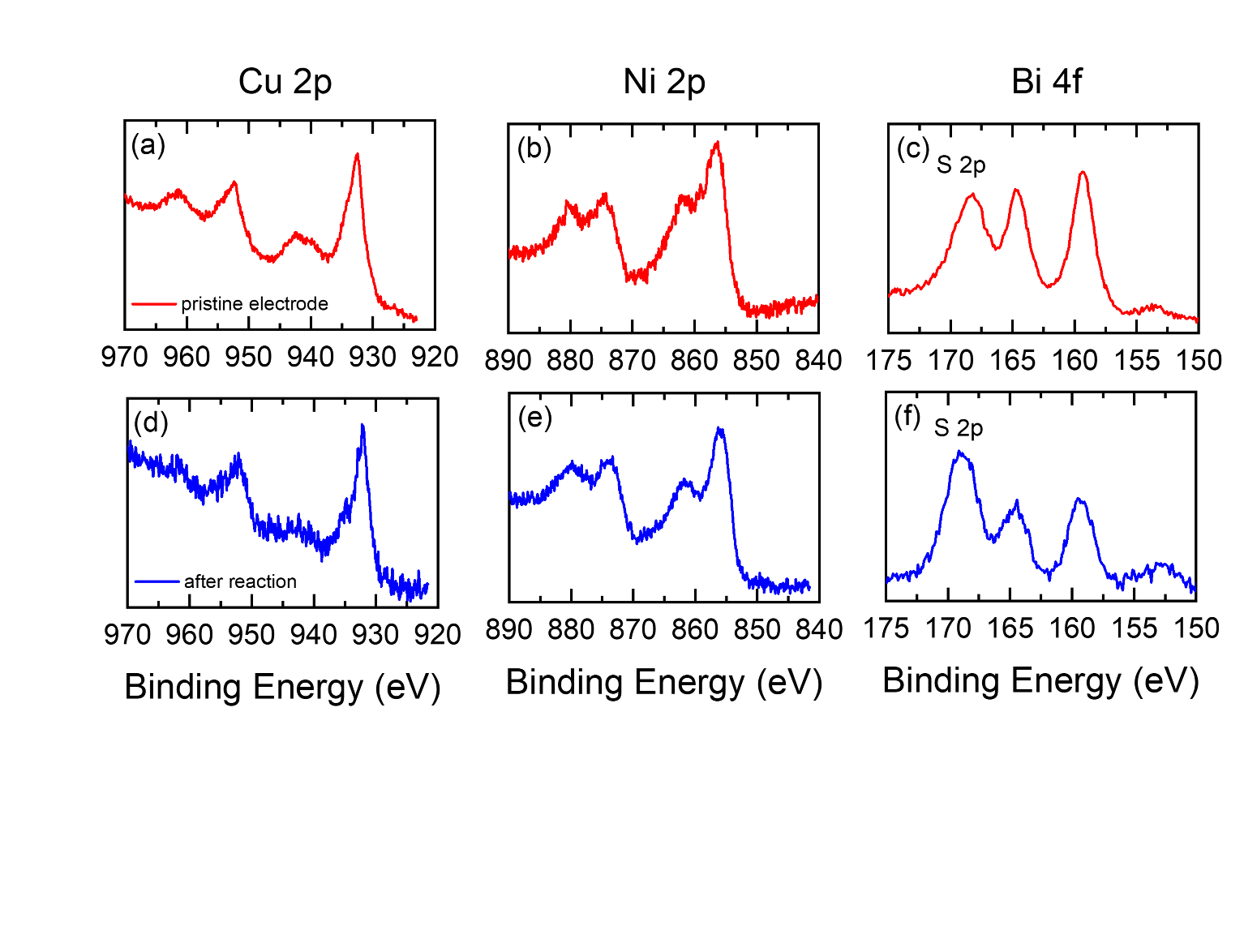


**Figure S23.** XPS spectra of CNBO on pristine electrode (**a, b, c**) and after one-hour reaction (**d, e, f**).

**Table S2**. XPS quantitative analysis of CNBO electrode before and after reaction.

|  | Cu at% | Ni at% | Bi at% |
| --- | --- | --- | --- |
| Pristine electrode | 65.0±17.5 | 30.5±7.6 | 4.6±0.9 |
| Electrode after reaction | 18.0±6.0 | 79.8±6.0 | 2.2±0.6 |


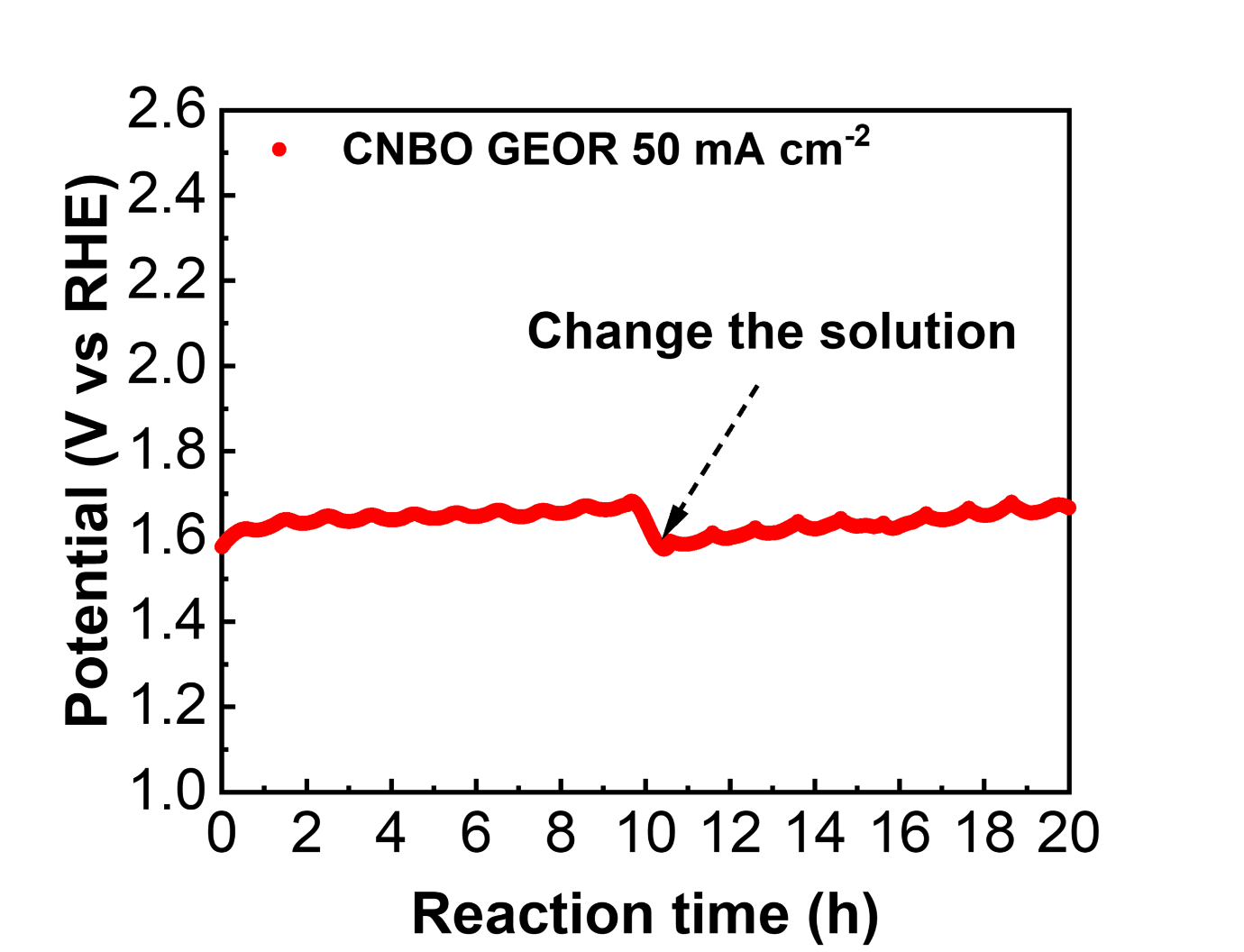


**Figure S24.** 20 hour-long CP scan recorded on CNBO at 50 mA cm^-2^, GEOR conditions.

**Table S3.** Time-dependent total Faradaic efficiency (*i.e.*, sum of all GEOR products FEs) and carbon balance obtained during the first 10 hours of the CP scan reported in **Figure S24**.

| **Time (h)** | **Charges passed (C)** | **Total Faradaic efficiency** | **Carbon balance (%)** |
| --- | --- | --- | --- |
| 1 | 45 | 100.3 | 100 |
| 2 | 90 | 103.8 | 98.2 |
| 3 | 135 | 101.7 | 99.4 |
| 4 | 180 | 100.9 | 99.5 |
| 5 | 225 | 99.1 | 99.3 |
| 6 | 270 | 96.1 | 99.1 |
| 7 | 315 | 98.2 | 99.9 |
| 8 | 360 | 94.9 | 98.4 |
| 9 | 405 | 96.8 | 99.5 |
| 10 | 450 | 91.4 | 96.7 |


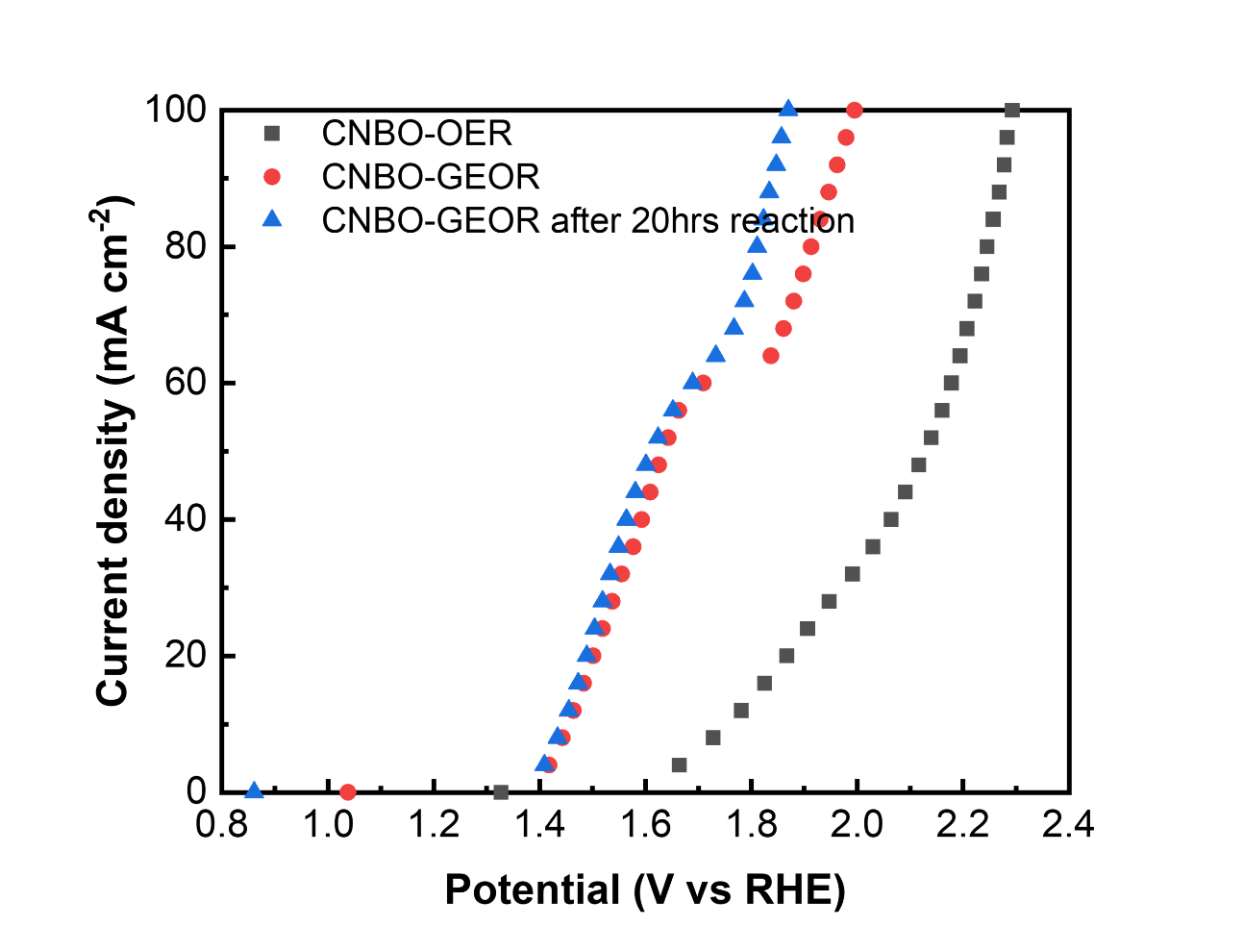


**Figure S25.** GLSVs traces recorded over CNBO before and after 20 hours-long electrolysis under GEOR conditions.

With the aim of assessing the structural and electrochemical stability of CNBO under industrially relevant conditions, we decided to move to high current and longer electrolysis time in a flow cell, namely 100 mA cm^-2^ for 100 hours in a two-compartment three-electrode PTFE-made flow cell (**Figure 3a**). An Anion Exchange Membrane (AEM, Fumasep FAB-PK-130, from FuelCellStore, activated according to the producer guidelines) separated the anodic and cathodic compartments. Hg/HgO and Ni foam was used as the reference and counter electrodes, respectively.

Due to the known phenomena of degradation/corrosion of carbon-based supports under alkaline and oxidative conditions (especially at high currents), we replace the anodic substrate with a metallic one, namely nickel foam (NF). Prior to use, NF was subsequently washed with acetone, 3 M HCl, and water under sonication for 15 minutes each. The CNBO/NF electrode was prepared as CNBO/carbon paper and the mass loading was equally set at 0.5 mg cm^-2^. Tests were then conducted on both the bare NF and CNBO/NF, in the presence and absence of glycerol, thus evaluating the blank and actual electrochemical performance, product distribution vs. time and stability. We note that the electrolyte has been replaced every 24 hours during long term stability tests, mainly to avoid glycerol depletion.

Linear sweep voltammetries (**Figure S26a**) was consistent with expectations. CNBO/NF displayed an earlier onset potential when glycerol is present into the electrolyte, highlighting the winning competition of GEOR over OER on this catalyst. Similarly, bare NF showed lower GEOR onset potentials in respect with OER ones. However, the OER and GEOR traces cross around 1.6 V vs RHE, indicating a winning OER competition for the bare substrate when operating at high current/voltage. Moving to long-term stability measurements (*i.e.*, long CP scans, **Figure 3b** in the main text), the (IR-corrected) potential needed to deliver a current density of 100 mA cm^-2^ over CNBO/NF is *ca*. 1.47 V vs RHE, which is *ca*. 120 mV lower than that of bare NF, consistently with the polarization curves. Both the substrate and the CNBO-modified electrode delivered stable electrochemical performances.

The product distribution yielded by both CNBO/NF and bare NF under GEOR conditions has been monitored in time by HPLC. Interestingly, the modest electrochemical performance of bare NF is accompanied by a low overall FE_GEOR_ (≈ 60%, **Figure S26c**). The GEOR products are formic (main product, FE_FA_ ≈ 35% in all samples), glycolic and glyceric acid as detected on CNBO in H-cell tests but with the additional presence of oxalic acid, most likely produced by glycolic acid oxidation through a secondary oxidation pathway (**Figure S15** and related discussion). Considering that the carbon balance reaches almost unity in all cases, we can assign the missing FE to O_2_ (OER competition). In contrast, CNBO/NF achieved ≈100% FE for GEOR, with FE for FA around 90% (**Figure 3c**). In full agreement with previous data and with the stability of the cathode potential observed by long CP scans, the product distribution on CNBO is constant over time. The voltammetric trace, collected on the electrode after the 100 hours long electrolysis (**Figure S26d**), further demonstrates the stability of CNBO/NF (the apparent increase in the performance might be assigned to a roughening/electrode area increase during operation).


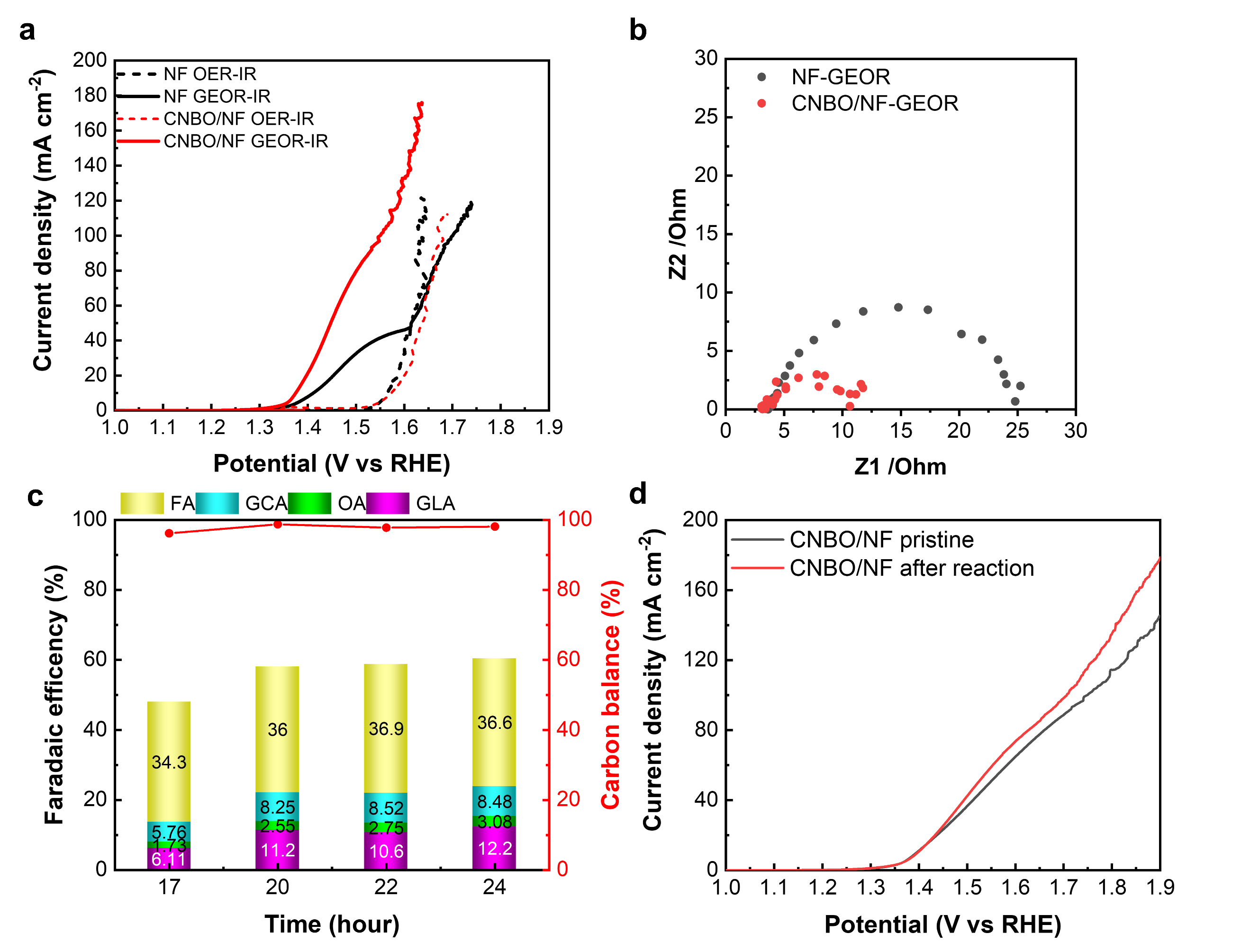


**Figure S26. (a)** Linear sweep voltammetric traces collected on CNBO/NF and NF, scan rate = 1 mVs^-1^; **(b)** galvanostatic electrochemical impedance spectroscopy obtained over NF and CNBO/NF; **(c)** related product distribution obtained over NF at 100 mA cm^-2^; **(d)** Linear sweep voltammetric traces collected on CNBO/NF before and after 100 hours of electrolysis. GLA: glyceric acid, GCA: glycolic acid, OA: oxalic acid, FA: formic acid.

Finally, the stability of the materials was then evaluated by structurally, morphologically, and compositionally characterizing post-reaction electrodes. SEM confirmed the retention of the structure of CNBO particle (**Figure S27a**). After 100-hour electrolysis,


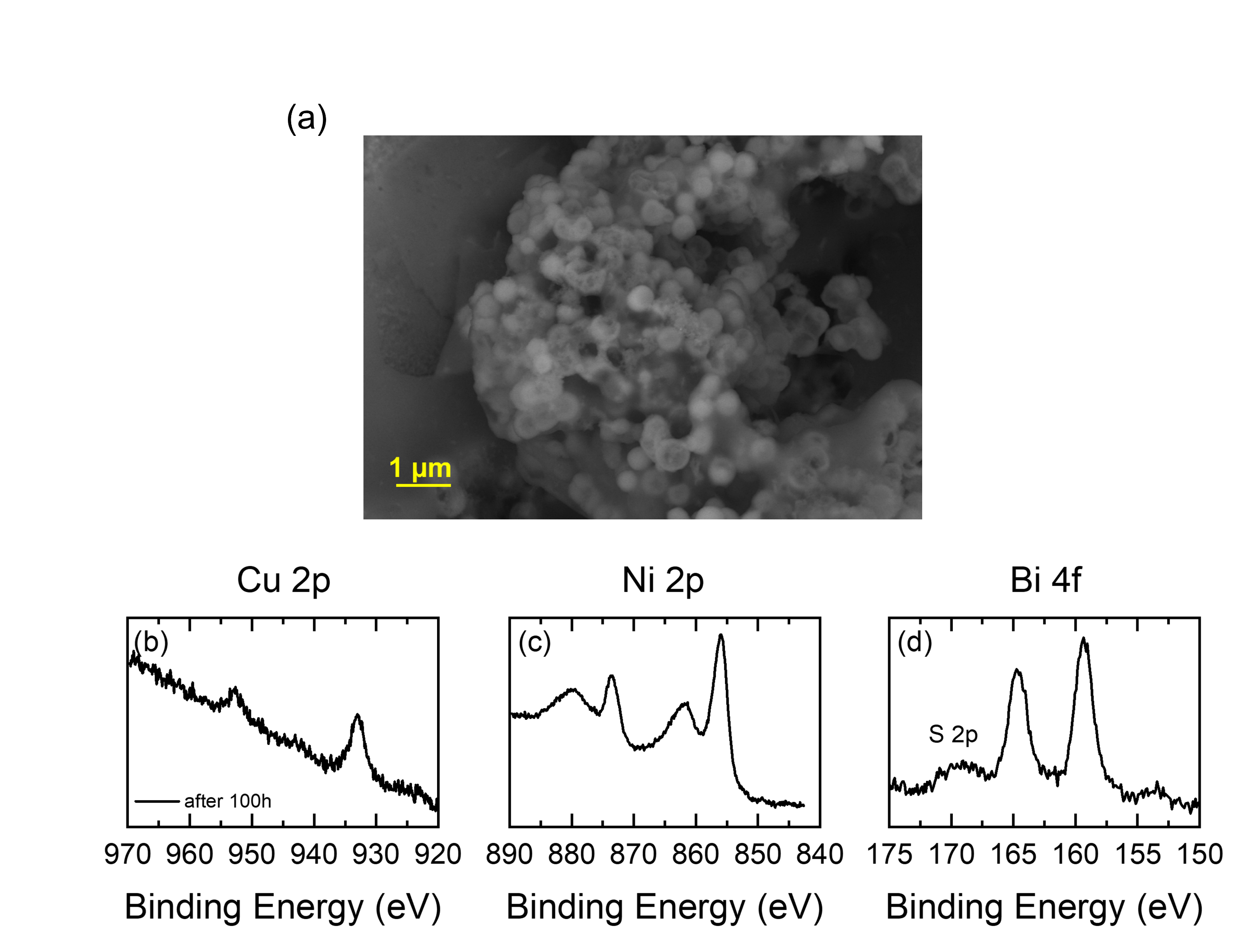


**Figure S27.** (**a**) SEM image, **(b)-(d)** XPS spectra of CNBO electrode after 100 hours of electrolysis.

**Table S4.** Performance comparison for representative electrocatalysts toward GEOR

| **Ni-based Catalyst** | **Electrolyte** | **Current Density**  mA cm^-^² | **Applied Potential**  V vs RHE | **Main Product** | **Total Faradaic Efficiency (FE)** | **Ref.** |
| --- | --- | --- | --- | --- | --- | --- |
| CNBO/CP | 1 M KOH + 0.1 M Glycerol | 50 | 1.58 | FA | 98% | This work |
| CNBO/NF | 1 M KOH + 0.1 M Glycerol | 100 | 1.47 | FA | 100% | This work |
| NiMoN/CFC | 0.1 M glycerol + 1 M KOH | 10 | 1.35 | FA | 97% | ^[10]^ |
| NiCrO/TM | 0.1 M glycerol + 1 M KOH | - | 1.45 | FA | 96% | ^[11]^ |
| NiCoOH/CC | 0.1 M glycerol + 1 M KOH | 100 | 1.58 | FA | ≈ 100 | ^[12]^ |
| Ni(OH)_2_/NF | 0.1 M glycerol + 2 M KOH | - | 1.5 | FA | 64 | ^[13]^ |
| NiO_x_MWCNTs-O_x_ | 1 M glycerol + 2 M KOH | ≈10 | 1.31 | FA | 96 | ^[14]^ |
| OX-Ni_x_Co_1-x_(OH)_2_/NF | 1.0 M KOH + 0.1 M glycerol | 10 | 1.3 | FA | 91 | ^[15]^ |
| Ni LDH/NF | 1.0 M KOH + 0.1 M glycerol | - | 1.55 | FA | 60 | ^[16]^ |
| PtSA-NiCo LDH/NF | 1.0 M KOH + 0.1 M glycerol | - | 1.38 | FA | 88.7 | ^[17]^ |
| E-NiV LDH/NF | 1.0 M KOH + 0.1 M glycerol | 10 | 1.23 | FA | - | ^[18]^ |
| A-NiFe LDH/NF | 1.0 M KOH + 0.1 M glycerol | 100 | 1.47 | FA | 99% | ^[19]^ |
| NiCo_2_O_4_/CFP | 1.0 M KOH + 0.1 M glycerol | 50 | 1.42 | FA | ≈55 | ^[20]^ |
| Ni-phen-NO_2_ catalyst/CFP | 2.0 M KOH + 0.1 M glycerol | - | 1.5 | FA | 92.7 | ^[21]^ |
| CoNiCuMnMo-NPs/CC | 1.0 M KOH + 0.1 M glycerol | 10 | 1.25 | FA | 90 | ^[22]^ |

# *In-situ* Raman spectroscopy analyses – Catalyst evolution under OER and GEOR conditions

Electrochemical *in-situ* Raman spectroscopy measurements were conducted using a Renishaw inVia Reflex Raman microscope with 633 nm laser excitation with a nominal power of 17 mW and an electrochemical workstation. The Raman cell was purchased from Redox.me Corp (https://redox.me/). The cell is made of PEEK with a quartz window between the sample and the objective. The spectrometer was equipped with a back-illuminated CCD detector and with an 1800 l/mm diffraction grating, giving a spectral resolution better than 1 cm^-1^. All the measurements were conducted in back-scattering geometry at room temperature. The samples were focused by a 50× long working distance objective providing a spot size of about one μm in diameter. The working electrode was inserted through the wall of the cell to align with the incident laser. A platinum wire and Ag/AgCl served as the counter and reference electrode, respectively. The electrolyte was continuously pumped to the cell with a flow rate of 1 mL min^-1^ using a double-headed peristaltic pump (MasterFlex L/S Intertek, Model No. 07528-30).


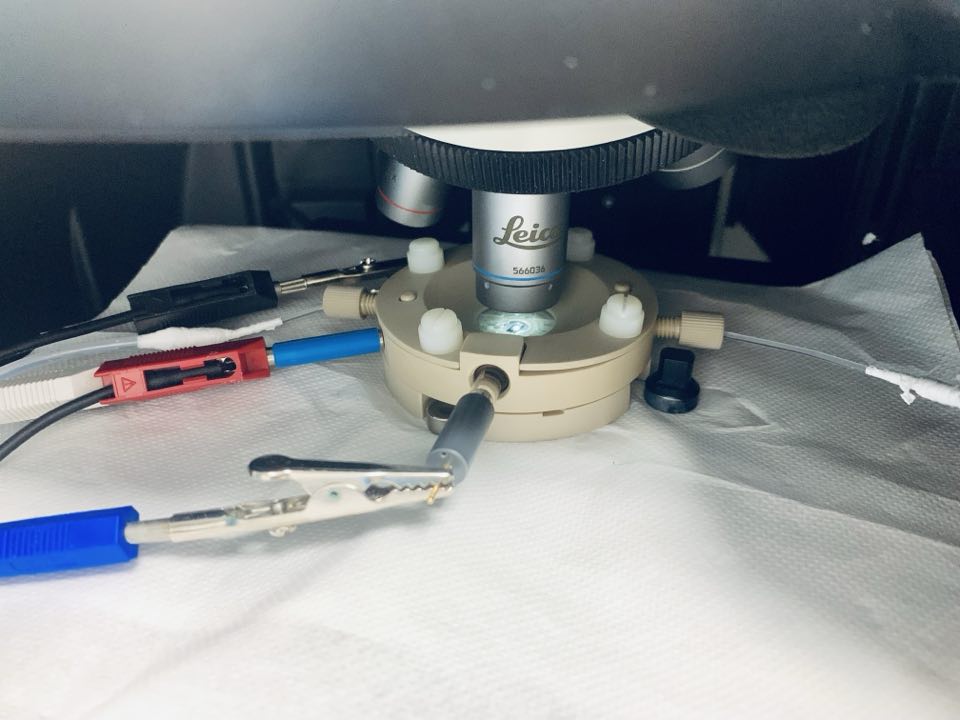


**Figure S28.** Picture of the *in-situ* Raman spectroscopy setup.

# GEOR + CO_2_RR coupling – Additional data

Full cell studies were carried out using the same flow cell as 100-hour stability test, with the addition of gas compartment assembled back side of the cathode. A two-electrode (full cell) configuration was used. In this setup, CNBO (electrode area equal to 0.5625 cm^-2^), Ag/PTFE or Sn/Sigracet 39BB (electrode area equals to 1.13 cm^-2^) were used as the working and counter electrodes, respectively. The electrolytes for OER and GEOR (25 mL in each reservoir, flow rate 6.5 mL min^-1^) were delivered by a double-headed peristaltic pump (MasterFlex L/S Intertek, Model No. 07528-30). CO_2_ was fed to the cathodes through the gas chamber with the rate of 10 mL min^-1^. As for H-cell testing, outlet gases were continuously conveyed to an inline GC.


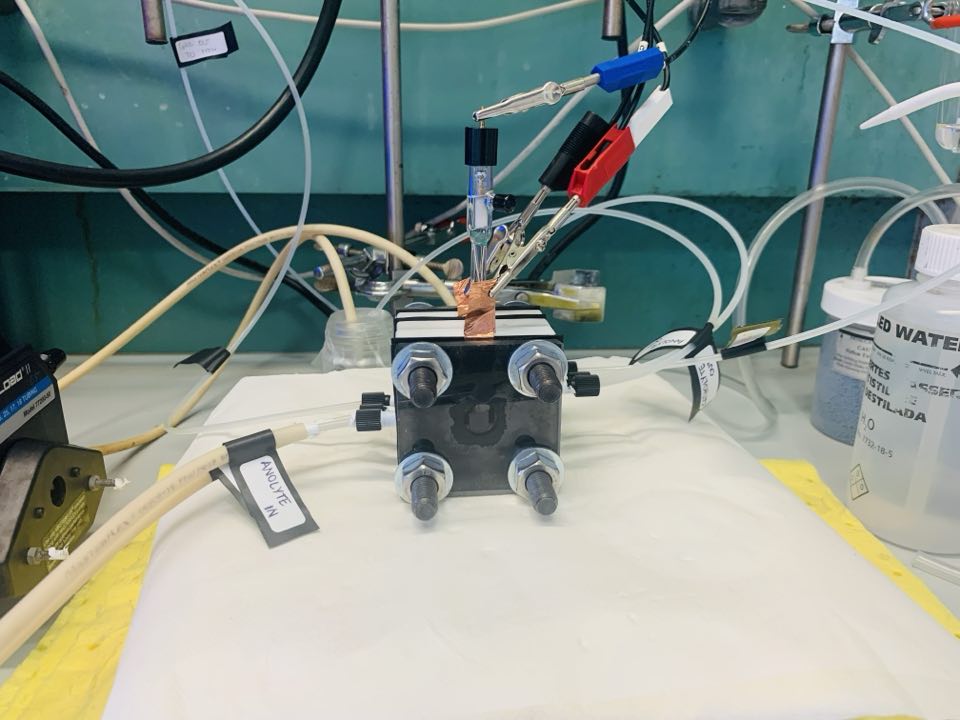


**Figure S29**. Picture of the electrochemical flow cell used for full cell studies coupling GEOR and CO_2_RR.


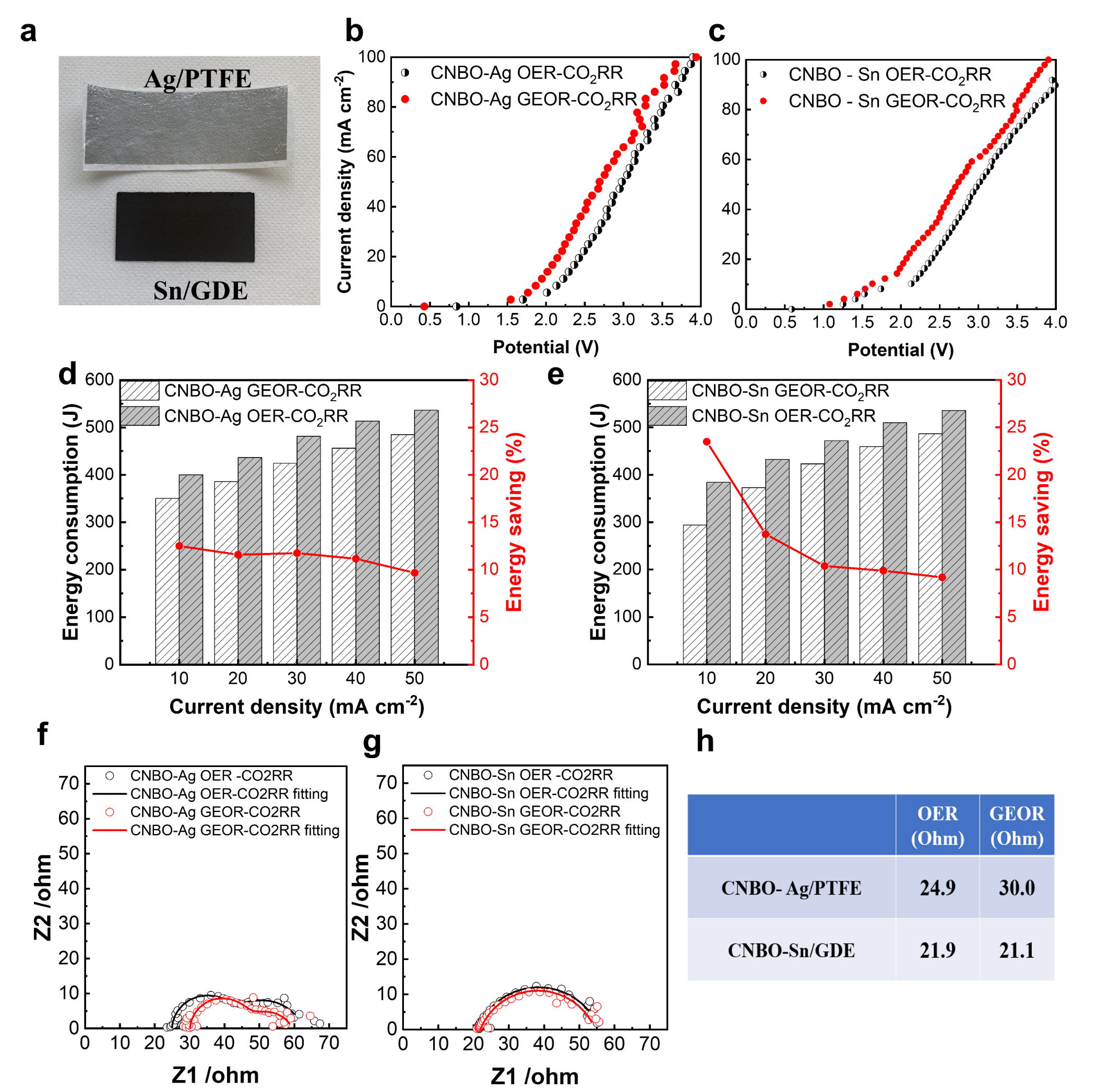


**Figure S30.** (**a**) Picture of Ag/PTFE and Sn/Sigracet 39BB cathodes; (**b-c**) GLSVs, (**d-e**) energy consumption-energy saving, (**f-g**) GEIS, and (**h**) system impedance values of OER and GEOR over CNBO coupling with CO_2_RR over Ag/PTFE and Sn/GDE.

# Supplementary Notes

## Supplementary Note 1

The “operational energy savings” (*i.e.*, reduction in the power consumption associated to the electrolysis) were calculated based on the difference between the total energy consumption of OER-CO_2_RR (E_OER-CO2RR_) and GEOR-CO_2_RR (E_GEOR-CO2RR_) full cell configurations. Therefore, the operational energy saving was determined by the following equation:

$Operational energy saving =\frac{E_{OER-CO2RR}- E_{GEOR-CO2RR}}{E_{OER-CO2RR}}\times$100%

in which E_OER-CO2RR_ and E_GEOR-CO2RR_ (Joule) were calculated based on the following equation:

$$E=I\times U\times t$$

Where I is the current (Ampere), U is cell potential (Volt) and t is the electrolysis time (second).

## Supplementary Note 2

It must be taken into account that electrons extracted from glycerol at the anode will travel to the cathode and participate in the formation of products via CO_2_RR. Therefore, here we introduce the concept of specific production energy, which is calculated based on the energy consumption over the amount of products generated by the full cell.

For convenience, we choose the setup of CNBO coupled with Sn/GDE for this evaluation, as both electrodes produced formic acid.

The ratio in the specific production energy required for formic acid in the two full cell configurations explored in this study can be calculated based on the following equation:

$$Specific production energy ratio =\frac{W_{E_{OER-CO2RR}}-W_{E_{GEOR-CO2RR}}}{W_{E_{OER-CO2RR}}}\times100\%$$

in which $W_{E_{OER-CO2RR}}$ and $W_{E_{GEOR-CO2RR}}$ represent for the energy consumed for for producing a mole of formic acid in OER-CO_2_RR and GEOR- CO_2_RR full cell configurations, respectively, and are calculated according to the following equations:

$W_{E_{OER-CO2RR}}=\frac{E_{OER-CO2RR}}{n_{FA(cathode)}}$; $W_{E_{GEOR-CO2RR}}=\frac{E_{GEOR-CO2RR}}{n_{FA}(whole cell)}$

in which n_FA(cathode)_ is the amount of formic acid produced at the cathode in the OER-CO_2_RR electrolyzer, n_FA(whole cell)_ is the amount of formic acid produced at both electrodes in the system of GEOR-CO_2_RR. Data used for these calculations are gathered in the following Table S5.

**Table S5.** Experimental data for energy-related calculations, collected in both OER-CO_2_RR and GEOR- CO_2_RR full cell configurations.

| **Reaction system** |  | **OER- CO_2_RR** | **GEOR-CO_2_RR** |
| --- | --- | --- | --- |
| Anode area | 0.5625 cm^-2^ |  |  |
| Cathode area | 1.13 cm^-2^ |  |  |
| Working current | 28.125 mA |  |  |
| Cell potential |  | 2.98 V | 2.55 V |
| Formic acid production after one hour of electrolysis (mmol) |  | Anode: N/A  Cathode: 0.425 | Anode: 0.340  Cathode: 0.425 |

Inserting Table S5 values into the previous equations, we obtain:

$$W_{E_{OER-CO2RR}}=\frac{E_{OER-CO2RR}}{n_{FA (cathode)}} =\frac{2.98\times28.125\times3600}{0.425\times{10}^{-3}}=709.94 J {mol}^{-1}$$

$$W_{E_{GEOR-CO2RR}}=\frac{E_{GEOR-CO2RR}}{n_{FA (whole cell)}} =\frac{2.55\times28.125\times3600}{0.765\times{10}^{-3}}=337.50 J {mol}^{-1}$$

According to the previous equation, the specific production energy of formic acid in the GEOR-CO_2_RR configuration is 52.46% lower when compared to the OER-CO RR one, as shown below:

$$Specific production energy ratio =\frac{W_{E_{OER-CO2RR}}-W_{E_{GEOR-CO2RR}}}{W_{E_{OER-CO2RR}}}\times100\%=\frac{709.94-337.50}{709.94}\times100\% =52.46\%$$

**Table S6.** Performance comparison of coupling GEOR and CO_2_RR.

| **System**  **Anode (A)**  **Cathode (C)** | **Electrolyte feed** | **Membrane** | **Applied J (mA cm^-2^)** | **Products**  **(Faradaic efficiency %)** | **Cell voltage(V)** | **Ref.** |
| --- | --- | --- | --- | --- | --- | --- |
| A: CNBO  C: Ag/PTFE | A: 1M KOH + 0.1 M Glycerol  C: 1 M KOH + CO_2_ gas | AEM | 50 | A: FA 88.1  C: CO 85.2 | 2.65 | This work |
| A: CNBO  C: Sn/GDE | A: 1M KOH + 0.1 M Glycerol  C: 1 M KOH + CO_2_ gas | AEM | 50 | A: FA 89.6  C: FA 85 | 2.55 | This work |
| A: CoP/NF  C: Ag/BOC-GDE | A: 1M KOH + 0.1 M Glycerol  C: CO_2_ - saturated 0.5 M KHCO_3_ | AEM | 50 | A: FA-  C: FA 98 | 2.2 | ^[23]^ |
| A: Ni_x_B  C: BiOBr -GDE | A: 1M KOH + 1 M Glycerol  C: 1 M KOH + CO_2_ gas | CEM | 50 | A: FA 45  C: FA 96 | 3.04 | ^[24]^ |
| A: Pt/C-PE  C: Bi-GDE | A: 1M KOH + 1 M Glycerol  C: KCl (0.5 M) + KHCO_3_ (0.45 M) | CEM | 200 | A: FA 23  C: FA 44 | 6.73 | ^[25]^ |
| A: Ni_3_S_2_-NF  C: In – Bi -GDE | A: KOH (1.0 M) + GLY (0.05 M)  C: KHCO_3_ (0.5 M) | BPM | 100 | A: FA 77-98  C: FA 79-83 | 5-6.3 | ^[26]^ |
| A: Pt/C-PE  C: Sn-GDE | A: KOH (1.0 M) + GLY (2.0 M)  C: KHCO_3_ (0.5 M) | CEM | 50 | A: FA 30  C: FA 74 | 4.4 | ^[27]^ |
| A: Ni-Co foam  C: Bi-C-GDE | A: KOH (1.0 M) + GLY (1.0 M)  C: 0.5 M KCl + 0.5 M KHCO_3_ (H_2_O/CO_2_) gas | Nafion | 45 | A: FA 55.4  C: FA 95.1 | 3.4 | ^[28]^ |
| A: Ni_0.33_Co_0.67_(OH)_2_@HOS/NF  C: BiOI/CP | A: KOH (1.0 M) + GLY (1.0 M)  C: KHCO_3_ 0.5 M | PEM | 22.4 | A: FA 90  C: FA 92 | 1.9 | ^[29]^ |

# Reference

[1] F. d. l. Peña1, , E. Prestat2, , V. T. Fauske, , P. Burdet3, , J. Lähnemann4, , P. Jokubauskas5, , T. Furnival6, , M. Nord7, , T. Ostasevicius8, , K. E. MacArthur9, , D. N. Johnstone, , M. Sarahan10, , J. Taillon11, , T. Aarholt12, , pquinn-dls, , V. Migunov13, , A. Eljarrat, , J. Caron, , C. Francis14, , T. Nemoto15, , T. Poon16, , S. Mazzucco, , actions-user17, , N. Tappy, , N. Cautaerts18, , S. Somnath, , T. Slater19, , M. Walls, , F. Winkler20, , H. W. Åne, hyperspy, **2022**.

[2] A. Wuttig, Y. Surendranath, *Acs Catal.* **2015**, *5*, 4479-4484.

[3] a) M. C. Haryanto, R. Hartanto, T.-G. Vo, C.-Y. Chiang, *J. Taiwan Inst. Chem. Eng.* **2023**, 105087; b) Y. Deng, A. D. Handoko, Y. Du, S. Xi, B. S. Yeo, *Acs Catal.* **2016**, *6*, 2473-2481; c) A. Sekkat, V. H. Nguyen, C. A. Masse de La Huerta, L. Rapenne, D. Bellet, A. Kaminski-Cachopo, G. Chichignoud, D. Muñoz-Rojas, *Commun. Mater.* **2021**, *2*, 78.

[4] a) V. Levitskii, V. Shapovalov, A. Komlev, A. Zav’yalov, V. Vit’ko, A. Komlev, E. Shutova, *Tech. Phys. Lett.* **2015**, *41*, 1094-1096; b) M. Umar, M. Y. Swinkels, M. De Luca, C. Fasolato, L. Moser, G. Gadea, L. Marot, T. Glatzel, I. Zardo, *Thin Solid Films* **2021**, *732*, 138763; c) J. Xu, W. Ji, Z. Shen, W. Li, S. Tang, X. Ye, D. Jia, X. Xin, *J. Raman Spectrosc.* **1999**, *30*, 413-415.

[5] J. Nai, Y. Tian, X. Guan, L. Guo, *J. Am. Chem. Soc.* **2013**, *135*, 16082-16091.

[6] W. Sun, N. Govindarajan, A. Prajapati, J. Huang, H. Bemana, J. T. Feaster, S. A. Akhade, N. Kornienko, C. Hahn, *ACS Appl. Mater. Interfaces* **2024**.

[7] M. Pourbaix, *NACE* **1966**.

[8] M. S. Houache, K. Hughes, R. Safari, G. A. Botton, E. A. Baranova, *ACS Appl. Mater. Interfaces* **2020**, *12*, 15095-15107.

[9] a) X. Cao, Y. Shi, W. Shi, G. Lu, X. Huang, Q. Yan, Q. Zhang, H. Zhang, *small* **2011**, *7*, 3163-3168; b) F. Chandoul, H. Moussa, K. Jouini, A. Boukhachem, F. Hosni, M. S. Fayache, R. Schneider, *J. Mater. Sci.: Mater. Electron.* **2019**, *30*, 348-358.

[10] Y. Li, X. Wei, L. Chen, J. Shi, M. He, *Nat. commun.* **2019**, *10*, 5335.

[11] Z. Xia, C. Ma, Y. Fan, Y. Lu, Y.-C. Huang, Y. Pan, Y. Wu, Q. Luo, Y. He, C.-L. Dong, *ACS Catal.* **2024**, *14*, 1930-1938.

[12] Z. He, J. Hwang, Z. Gong, M. Zhou, N. Zhang, X. Kang, J. W. Han, Y. Chen, *Nat. Commun.* **2022**, *13*, 3777.

[13] J. Wu, J. Li, Y. Li, X. Y. Ma, W. Y. Zhang, Y. Hao, W. B. Cai, Z. P. Liu, M. Gong, *Angew. Chem. Int. Ed.* **2022**, *61*, e202113362.

[14] D. M. Morales, D. Jambrec, M. A. Kazakova, M. Braun, N. Sikdar, A. Koul, A. C. Brix, S. Seisel, C. Andronescu, W. Schuhmann, *ACS Catal.* **2022**, *12*, 982-992.

[15] Y. Zou, W.-D. Zhang, H. Xu, J. Yang, J. Liu, Z.-G. Gu, X. Yan, *J. Colloid Interface Sci.* **2023**, *650*, 701-709.

[16] D. Kim, C. Dang Van, M. S. Lee, M. Kim, M. H. Lee, J. Oh, *ACS Catal.* **2024**, *14*, 7717-7725.

[17] H. Yu, W. Wang, Q. Mao, K. Deng, Z. Wang, Y. Xu, X. Li, H. Wang, L. Wang, *Appl. Catal. B: Environ.* **2023**, *330*, 122617.

[18] L. Dong, G.-R. Chang, Y. Feng, X.-Z. Yao, X.-Y. Yu, *Rare Metals* **2022**, 1-12.

[19] Y. Song, X. Wan, Y. Miao, J. Li, Z. Ren, B. Jin, H. Zhou, Z. Li, M. Shao, *Appl. Catal. B: Environ.* **2023**, *333*, 122808.

[20] Y. Duan, M. Xue, B. Liu, M. Zhang, Y. Wang, B. Wang, R. Zhang, K. Yan, *Chin. J. Catal.* **2024**, *57*, 68-79.

[21] J. Wu, X. Liu, Y. Hao, S. Wang, R. Wang, W. Du, S. Cha, X. Y. Ma, X. Yang, M. Gong, *Angew. Chem. Int. Ed.* **2023**, *62*, e202216083.

[22] L. Fan, Y. Ji, G. Wang, J. Chen, K. Chen, X. Liu, Z. Wen, *J. Am. Chem. Soc.* **2022**, *144*, 7224-7235.

[23] X. Guo, S.-M. Xu, H. Zhou, Y. Ren, R. Ge, M. Xu, L. Zheng, X. Kong, M. Shao, Z. Li, *ACS Catal.* **2022**, *12*, 10551-10559.

[24] J. R. Junqueira, D. Das, A. Cathrin Brix, S. Dieckhöfer, J. Weidner, X. Wang, J. Shi, W. Schuhmann, *ChemSusChem* **2023**, *16*, e202202349.

[25] K. Fernández-Caso, A. Pena-Rodríguez, J. Solla-Gullón, V. Montiel, G. Díaz-Sainz, M. Alvarez-Guerra, A. Irabien, *J. CO2 Util.* **2023**, *70*, 102431.

[26] B. van den Bosch, B. Rawls, M. B. Brands, C. Koopman, M. F. Phillips, M. C. Figueiredo, G. J. M. Gruter, *ChemPlusChem* **2023**, *88*, e202300112.

[27] J. Vehrenberg, J. Baessler, A. Decker, R. Keller, M. Wessling, *Electrochem. commun.* **2023**, *151*, 107497.

[28] K. Fernández-Caso, M. Molera, T. Andreu, J. Solla-Gullón, V. Montiel, G. Díaz-Sainz, M. Álvarez-Guerra, A. Irabien, *Chem. Eng. J.* **2024**, *480*, 147908.

[29] Y. Pei, Z. Pi, H. Zhong, J. Cheng, F. Jin, *J. Mater. Chem. A* **2022**, *10*, 1309-1319.
